# Chromatin Landscape Dictates HSF Binding to Target DNA Elements

**DOI:** 10.1371/journal.pgen.1001114

**Published:** 2010-09-09

**Authors:** Michael J. Guertin, John T. Lis

**Affiliations:** Department of Molecular Biology and Genetics, Cornell University, Ithaca, New York, United States of America; National Cancer Institute, United States of America

## Abstract

Sequence-specific transcription factors (TFs) are critical for specifying patterns and levels of gene expression, but target DNA elements are not sufficient to specify TF binding in vivo. In eukaryotes, the binding of a TF is in competition with a constellation of other proteins, including histones, which package DNA into nucleosomes. We used the ChIP-seq assay to examine the genome-wide distribution of *Drosophila* Heat Shock Factor (HSF), a TF whose binding activity is mediated by heat shock-induced trimerization. HSF binds to 464 sites after heat shock, the vast majority of which contain HSF Sequence-binding Elements (HSEs). HSF-bound sequence motifs represent only a small fraction of the total HSEs present in the genome. ModENCODE ChIP-chip datasets, generated during non-heat shock conditions, were used to show that inducibly bound HSE motifs are associated with histone acetylation, H3K4 trimethylation, RNA Polymerase II, and coactivators, compared to HSE motifs that remain HSF-free. Furthermore, directly changing the chromatin landscape, from an inactive to an active state, permits inducible HSF binding. There is a strong correlation of bound HSEs to active chromatin marks present *prior* to induced HSF binding, indicating that an HSE's residence in “active” chromatin is a primary determinant of whether HSF can bind following heat shock.

## Author Summary

Many Transcription Factors (TFs) have been shown to bind DNA in a sequence-specific manner. However, only a sub-set of possible binding sites are occupied in vivo, and it remains unclear how TFs discriminate between sequences of equal predicted binding affinity. We set out to determine how a specific TF, Heat Shock Factor (HSF), distinguishes between utilized and unused potential binding sites. HSF is uniquely qualified to study this problem, because HSF is inactive and lowly bound to DNA in unstressed cells and upon stress HSF becomes active and strongly binds to DNA. We compared the properties of the unstressed chromatin between the sites that become HSF-bound or remain HSF-free following stress activation. We find that sites that are destined to be bound strongly by HSF after stress are associated with distinct chromatin marks compared to sites that are unoccupied by HSF after heat shock. Furthermore, chromatin landscape can be changed from a restrictive to a permissive state, allowing inducible HSF binding. These finding suggest that TF binding sites can be predicted based on the chromatin signatures present prior to induced TF recruitment.

## Introduction

Signal-dependent activation of transcription is a highly regulated process that is dictated by transcriptional activators that selectively identify and function at sequence-specific DNA motifs. The most basic function of sequence specific activators is to discriminate between binding sites in the context of the entire genome [1]–[4], but the mechanism by which this occurs is poorly understood. Two main mechanisms have been proposed that explain the observed in vivo binding specificity (reviewed in [5]): TFs are occluded from cognate site by chromatin structure or TF binding is facilitated by cooperative interactions with cofactors. In vivo, TFs are in competition with chromatin factors, which may limit TF access to cognate binding sites [6], [7]. Early sequence-specific ChIP experiments of homeoproteins revealed that binding sites are preferentially accessible if target motifs are located within active genes [1]. More recently, advances in genome-wide characterization of histone modifications and chromatin structure have begun to identify additional requirements for the binding of TFs. In human cells, it has been shown that the H3K4me1 and H3K4me3 modifications are present at inducible STAT1 binding sites prior to interferon-gamma stimulation [8]. In *Drosophila*, H3K36me3 has been revealed as an important histone mark for male-specific lethal (MSL) complex binding [9], [10]. However, the Hox proteins primarily discriminate between equivalent predicted binding sites by cooperative interactions with DNA-bound cofactors (reviewed in [11]). These findings indicate that the binding of TFs depend upon the chromatin landscape as well as specific sequence elements, and we set out to determine the extent to which chromatin affects TF binding genome-wide. Characterizing the mechanistic parameters by which TFs locate and bind to target DNA sequences will provide insight into a critical early step in a cell's ability to orchestrate patterns of gene expression in response to developmental, nutritional, and environmental signals.

Heat Shock Factor (HSF) has a conserved function as the master regulator of the heat shock (HS) response from organisms as distantly related as yeast and humans [12]. The HS genes of *Drosophila melanogaster* are an attractive model system to study the general functions of HSF and its induced transcriptional regulation [13]. HSF is present as a nuclear-localized monomer during non-stress conditions [14]; upon stress, HSF homotrimerization [15] mediates binding to HSF Sequence-binding Element (HSE) motifs within seconds [16], [17], which strongly activates a set of HS genes. While transcription factor binding to DNA is necessary for *cis* regulation of target genes, not all TF binding is necessarily functional [18]. For instance, HSF has been mapped to over 164 cytological sites on the polytene choromosomes of *Drosophila* salivary gland cells after HS [19], but only 9 cytological loci exhibit HS-induced transcription elongation factor recruitment and activation [20]–[23]. It remains unclear how HSF discriminates between sites and selectively stimulates functional gene activation.

In this study, we set out to determine the comprehensive set of HSF binding sites in the *Drosophila* genome and the molecular basis for the binding. We used ChIP (chromatin immunoprecipitation) followed by sequencing [24], adapted for high throughput detection (ChIP-seq) [25]–[27], to map the sites of HSF binding in an unbiased manner with high sensitivity and resolution. We made use of the ChIP-chip datasets from the model organism ENCyclopedia Of DNA Elements (modENCODE) consortium [28], [29], which profiles histone modifications, histone variants [30], insulators [31], [32], and Pol II. These datasets describe critical features of the chromatin landscape in unstressed cells. Using this data, we contrasted the chromatin landscape before HS induction at induced HSF-bound HSE motifs and HSE motifs that remain HSF-free. The roles of many of the modENCODE chromatin features are well established [30]–[33], thus the absence or presence of one or many of these features provides insight into the mechanism of HSF binding.

## Results

### ChIP-seq in HSF depleted cells is a critical control for optimizing sensitivity and specificity

**Figure 1:**
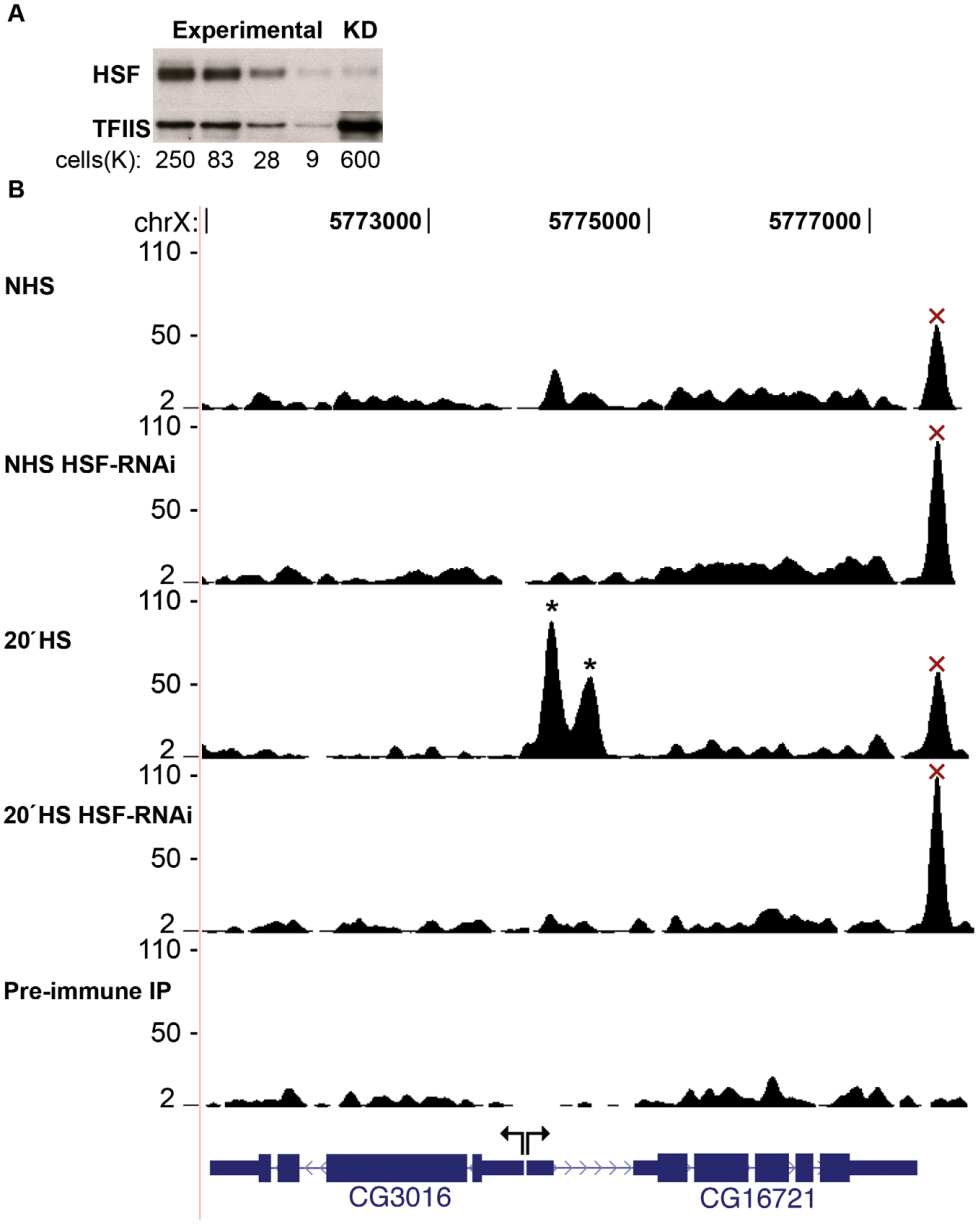
HSF depletion filters false positive peaks. (A) Densitometry of the loading control (TFIIS) confirmed that the intensity of each band was proportional to the number of cells loaded. The HSF-KD HSF band is 1.6 times the intensity of the most dilute HSF band of the standard curve, indicating a 40-fold depletion of HSF. (B) The UCSC Genome Browser is used to show a locus that contains two legitimate HS inducible/HSF-RNAi-sensitive binding sites (represented by asterisks) and a false-positive peak that is neither inducible nor sensitive to HSF depletion (represented by “×”). The y-axis scale is linear (from 2 to 110) and normalized for each experiment (shifted tags/10 bp/10 million sequences in the library). Mock IP with the pre-inoculated animal serum served as a background dataset (Pre-immune IP).

To determine the comprehensive set of HSF binding sites, we performed two highly correlated, independent ChIP-seq experiments in *Drosophila* S2 cells [34] for both non-heat shock (NHS) and 20′ HS conditions (Figure S1). We used well-characterized ChIP-grade HSF antiserum [23], [35] which specifically recognizes one HSF-RNAi sensitive Western blot band from whole S2 cell extract (Figure 1A) [35] and generates the expected global HSF-binding pattern observed by indirect immunofluorescence (IF) polytene staining [19], [36], [37]. Despite the specificity observed in these assays, we set out to directly assess specificity in genome-wide ChIP by identifying any HSF-non-specific DNA pull-down. We performed two independent HSF antiserum ChIP-seq control experiments, for each condition (NHS and 20′ HS), in cells that were depleted of HSF by RNAi. This approach approximates a control immunoprecipitation (IP) from cells that lack the factor of interest [38], [39].

HSF-knock down (KD) depleted endogenous levels of HSF to less than 2.5% of control cells as measured by quantitative Western blot (Figure 1A). Importantly, the level of HSF in RNAi depleted cells was reduced at the promoters of well-characterized HS genes, including the highest affinity *Hsp83* promoter (Figure S2). Due to the unique presence of tandem HSEs and cooperative HSF binding, the in vitro dissociation constant for the HSF/*Hsp83* promoter interaction is on the order of single-digit femtomolar [40], and the *Hsp83* promoter harbors the only strongly bound sites during NHS [19] (Figure S1). Since our KD of HSF was successful at reducing HSF levels at the highest affinity binding site, the signal intensity of all HSF-specific peaks should be susceptible to HSF-RNAi depletion as well. Therefore, we discarded peaks that were resistant to HSF depletion, as these are very likely false positives (Figure 1B, Figure S3, Figure S4 and Materials and Methods).

Our analysis of the ChIP-seq data aimed to increase the sensitivity of HSF detection without compromising confidence. To this end, we relied upon two peak calling programs [41], [42] to determine HSF binding sites (see Materials and Methods and Figure S3). Lower confidence peaks were initially considered and later filtered out if found resistant to HSF-RNAi. We detected 464 HSF-specific peaks after 20′ of HS (Dataset S1). We recovered 118 RNAi-sensitive peaks that would have otherwise been discarded because of high false discovery rates (FDR) (Figure S3). In addition, we filtered out 310 non-specific peaks that had FDRs below 0.1 (Figure S3), because they were completely insensitive (and actually increased in intensity) to HSF-KD and exhibit comparable NHS intensity (Figure 1B). Therefore, performing ChIP-seq in cells that were depleted of HSF by RNAi increased the sensitivity and specificity of peak calling.

### HSF inducibly binds to a specific consensus motif

**Figure 2:**
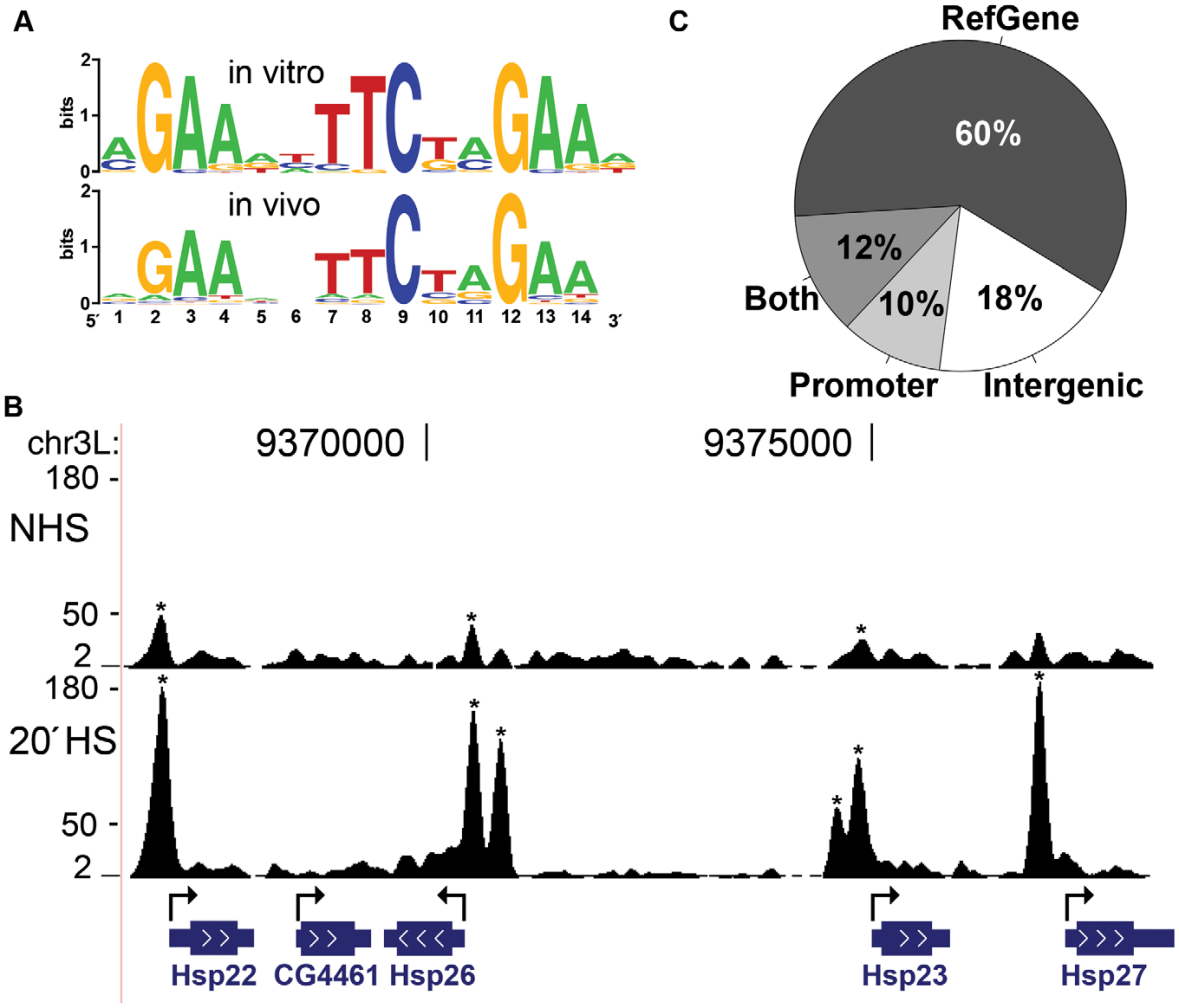
Characterization of HSF binding sites. (A) The PSWM derived from in vitro band shift assays [16] (top) and this study (bottom) are compared. Sequence logos were generated using WebLogo [93]. (B) The 67B locus harbors known heat shock protein (hsp) genes. The y-axis scale is linear (from 2 to 180) and directly comparable for each condition (shifted tags/10 bp/10 million sequences in the library). HSF binding sites, detected by our peak calling criteria (asterisks), increased in signal intensity or appeared de novo as cells were shifted from NHS to HS. (C) HSF binding sites are found within the body of RefGenes (72%: 316 sites), in the 500 bases upstream of TSS (22%: 97 sites), and within intergenic regions (18%: 81). A precise genomic sequence can be *both* within a gene and within the promoter of an upstream gene; 52 binding sites (the 12% slice) fall in this category.

We derived a position-specific weight matrix (PSWM) [43] and generated an in vivo composite HSF binding site using all 464 HSF peaks occupied after 20′ HS (Figure 2A bottom). Greater than 95% (442/464) of the peaks contained at least one HSE (Figure 2A bottom) with a p-value below 0.001 (Figure S4 and Materials and Methods), indicating that we are primarily detecting HSF directly bound to DNA. In contrast, the distribution of HSE motifs surrounding the HSF-RNAi resistant peaks approximates random expectation (Figure S4). This analysis indicates that the majority of RNAi resistant peaks are false positives that likely result from antiserum cross-reaction with another DNA binding protein, as these peaks are not present in the pre-immune IP. Consistent with the high affinity motif derived by in vitro band shift assays [16] (Figure 2A top), the in vivo HSE is a tandem array of three oppositely oriented five base pair units: AGAAN. In vitro HSF can bind to elements containing three five base pair units, regardless of their orientation relative to one another—although the opposite orientation of three 5 base pair units bound more tightly than direct repeats [16]. Our ChIP-seq study reveals that the opposite orientation of the tandem 5 base pair units is absolutely critical for detectable binding in vivo.

At those peaks that contain HSE motifs, we inferred the HSF binding sites at base pair resolution using the consensus-binding motif derived from this study (Figure 2A bottom). If multiple HSEs were within the 442 HSE containing peaks, the motif closest to the peak center was scored as the HSF binding site (Dataset S2). Our analysis recovered all previously well-characterized HSF binding sites within the promoters of HS responsive genes (Figure 2B, Figure S2, and Dataset S3), including the multi-copy *Hsp70* gene (Figure S5). We found that only 20 of the high-confidence HS peaks are detected during NHS conditions, and with a much lower density of tag counts (Figure 2B). Despite the fact that a corresponding NHS peak could not be detected at 422 of the 442 HS peaks, sequence tags are associated with these regions and signal may be above background, but below our threshold for detecting peaks. We considered that true signal should still be susceptible to HSF-KD (Figure S6) and concluded that the majority of these 422 sites are either completely devoid of HSF or contain extremely low, thus undetectable, levels of HSF under NHS conditions. Taken together, our analysis reveals that HSF behaves as we expected from previous molecular analyses of particular genes [16], [17] and from comprehensive, but low resolution, cytological analyses [19]: HSF binds strictly to HSEs and these sites are absent or show drastically reduced occupancy during NHS conditions.

Previous independent reports indicate that ChIP signal intensity positively correlates with motif conformity [3], [4], [44]. We find, however, that HSF binding sites conforming more stringently to the PSWM contain a comparable density of sequence tags as degenerate HSF binding sites (Figure S7A and S7B), suggesting that sequence alone is not driving HSF binding affinity.

### HSF binds to only a fraction of potential motifs in vivo

Although bona fide HSF binding sites contain highly specific HSE motifs, only a small fraction of potential HSE motifs are occupied by HSF. To search for HSF-free binding sites, we employed a conservative cut-off for conformity to the consensus HSE by using a p-value of 5×10^−6^ or less [43], while ensuring that the flanking region is mappable [45]. There are 708 HSF-free motifs (Dataset S4 and Figure S8A) that meet these criteria. Less than 15% (107/815) of the mappable HSE motifs with a p-value of 5×10^−6^ or less are detectably bound by HSF after HS. Upon closer inspection (Figure S6), we find that HSF-free motifs are absolutely HSF-free during NHS, and these same motifs are either unoccupied or infrequently occupied after HS. In contrast, HSF-bound motifs are either very weakly occupied or unoccupied prior to HS, and show strong inducible binding after HS induction. Therefore, these two categories of motifs, HSF-free and HSF-bound, are distinct from one another and are compared below.

We determined the distribution of HSF binding sites relative to annotated genes and promoter regions. Annotated genes account for 60.6% of the *Drosophila* reference genome (Figure S8B), however, 72% of the HSF-bound motifs are found within gene boundaries (Figure 2C). HSF-bound motifs within promoters (500 bp upstream of a transcription start site (TSS)) were also enriched, accounting for 22% of the total bound motifs (Figure 2C), while such promoter regions only account for 3.4% of the total reference genome (Figure S8B). In contrast, the classification of the 708 HSF-free motifs is much closer to a background distribution; 63% HSF-free motifs are within genes and 5.5% are within promoters (Figure S8A). These results indicate that HSE motifs are not simply enriched within gene and promoter boundaries, but that HSF preferentially interacts with HSEs that are present within genes and promoters.

### HSF discriminates between HSEs based on local signatures of active chromatin

We hypothesized that HSF discriminates between equivalent HSE sequences in vivo based on the chromatin landscape in which motifs reside. Previous work shows that HSF preferentially binds acetylated nucleosomes in vitro and more recently that the androgen receptor preferentially binds nucleosomes modified with methylated H3K4 in vivo [46], [47]. To determine the extent to which HSF binding is influenced by chromatin in vivo, we compared the NHS chromatin state between the motifs that become HSF-bound or remain HSF-free following HS, excluding the 20 HSF-bound motifs in which HSF was detected during NHS (Dataset S5). Using modENCODE S2 ChIP-chip data [28]–[32], we examined the composite intensity of microarray signal in the region surrounding each HSE. We found that HSF-bound motifs were generally associated with marks of active chromatin, even though these modENCODE signals were generated under NHS conditions (Figure S9). The HSF-free motifs, as a class, were neither enriched nor depleted for any particular factor, histone modification, or histone variant.

**Figure 3:**
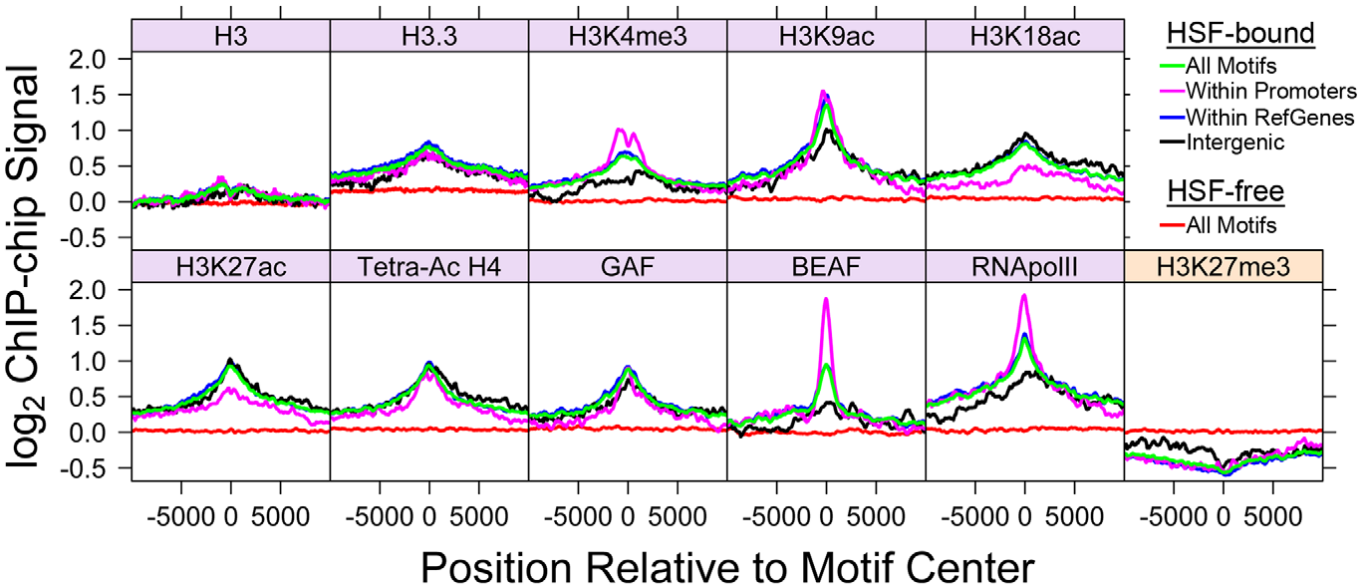
Bound HSE motifs contain marks of active chromatin prior to HSF binding. The average factor or histone modification occupancy was assigned in 100 base windows (step size of 50) around HSF-free HSE motifs (red) and HSF-bound HSE motifs (green). HSF-bound motifs are categorized by annotation class: motifs within promoters (magenta), RefGene bodies (blue), and intergenic regions (black). Canonical active chromatin marks are enriched at HSF-bound motifs (purple). H3K27me3 is depleted at HSF-bound motifs (orange).

Nucleosome occupancy of potential TF binding sites generally restricts TF binding [6], [7], so we examined the distribution of histones and histone variants around HSF motifs. We expected the HSF-bound motifs to be depleted of nucleosomal H3. The composite profiles show that nucleosomal H3 is clearly not depleted (Figure 3); in fact, we observe a slight increase in H3 levels at bound HSEs compared to free HSEs. This observation is in contrast to the general inhibitory nature of nucleosomes and the previous view of HSF binding, as the small set of well-characterized HSF binding sites are devoid of canonical nucleosomes prior to HS [48], [49]. The histone variant H3.3, which associates with active genes [30], displays a peak centered on the HSE motif (Figure 3). These results indicate that HSF binding specificity is not simply dictated by nucleosome-free DNA sequence.

In recent years, considerable attention has focused on the plethora of covalent histone modifications that occur on the N-terminal tails of histones, the enzymes responsible for catalyzing histone modifications, and the functional consequence of each modification. Acetylation of histone residues H3K9, H3K18, H3K27, H4K5, H4K8, and H4K16 were found to associate with HSF-bound motifs (Figure 3 and Figure S10). Each one of these acetylation marks has previously been shown to mark active chromatin [33], [50]. We find that the methylation marks H3K4me3 and H3K79me2, which associate with active genes [33], [51], are also enriched around the HSF-bound HSEs (Figure 3 and Figure S10). Mono-ubiquitylation (Ub) of H2B, a modification that is necessary for methylation of H3K4 [52], correlates with HSF-bound motifs as well (Figure S10). Conversely, marks of repressive chromatin, H3K27me3 and H3K9me3, were found to be depleted or at background levels (Figure 3 and Figure S10).

We considered that HSEs and histone marks cooperate to specify HSF binding. Transcription factors can bind acetylation and methylation marks through specific domains such as bromodomains, chromodomains and PHD domains (reviewed in [53]). For example, the MSL complex harbors a chromodomain, accounting for preferential recognition and binding of the H3K36me3 mark in *Drosophila* [9]. Interestingly, the HSF protein is devoid of all of these domains, and thus cannot be binding to DNA and histone methyl or acetyl marks cooperatively by any of these well-characterized interactions.

Comparison of HSF-bound motifs with TF binding data reveals that HSF co-localizes with factors that are associated with active transcription. The presence of Pol II is the foremost indicator of an active gene or a gene that is primed to be activated. The composite Pol II profile at HSF-bound HSEs exhibits a striking peak, even in instances where bound HSEs are within intergenic regions (Figure 3). Likewise, we observe a strong BEAF (boundary element-associated factor) signal centered on HSF-bound motifs (Figure 3). BEAF is an insulator that localizes to transcriptionally active and paused polymerase-harboring genes [32]. The multifaceted TF, GAGA Associated Factor (GAF), is associated with both paused polymerases and HSF-bound motifs [54] (Figure 3). Taken together, these profiles indicate that HSF binds to sites that contain hallmarks of open and active chromatin.

**Figure 4:**
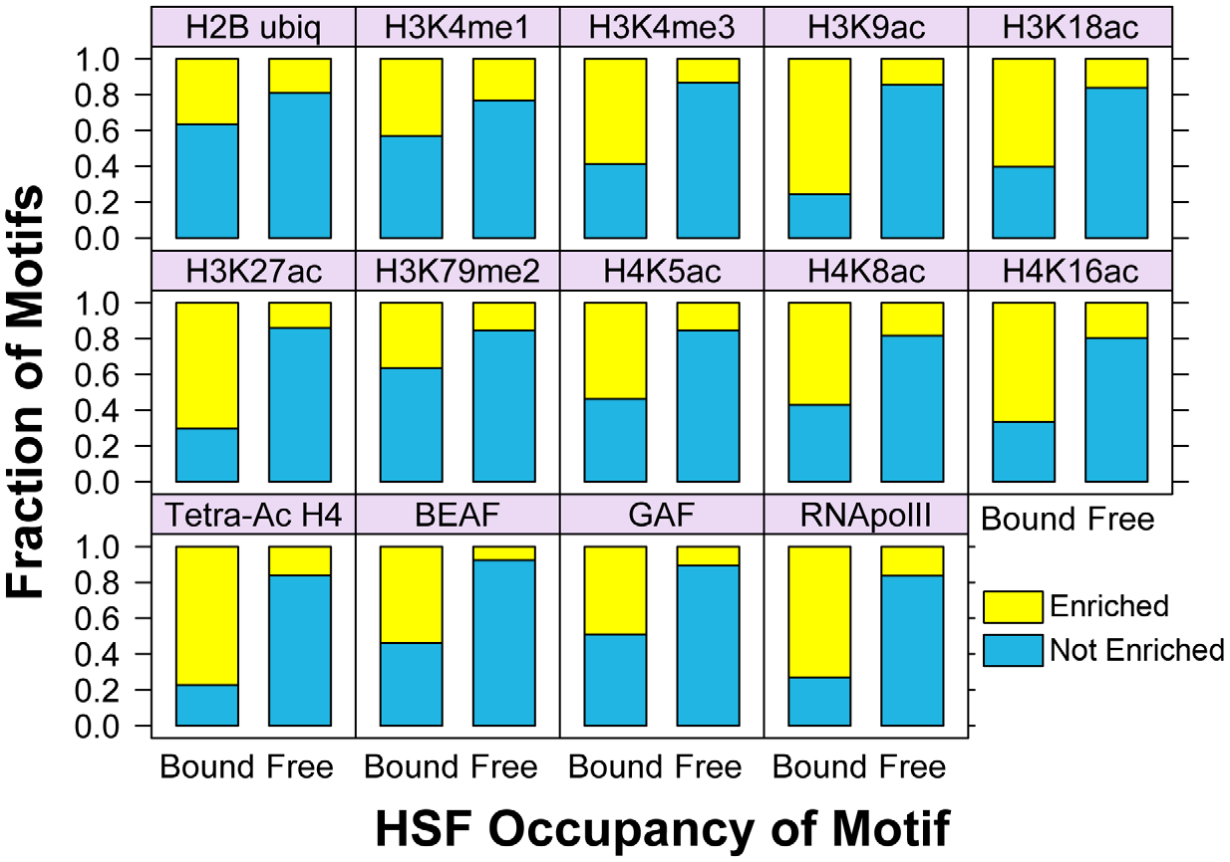
Bound HSE motifs are statistically associated with marks of active chromatin, compared to HSF-free motifs. For each factor shown, the Fisher exact test was used to determine the statistically significant association of HSF-bound motifs (left bar in each panel) with each modENCODE factor or histone modification, compared to HSF-free motifs (right bar). The yellow fraction of the bar chart represents HSF binding sites that are within regions of significant enrichment, while blue depicts all non-enriched sites.

These composite profiles provide an average view of HSF-binding, which could potentially be influenced by a small population of binding sites. We used the available “Regions of Significant Enrichment” tracks from modENCODE to determine which motifs (HSF-bound or HSF-free) were present within the significantly enriched regions of a given factor or modification. We employed the Fisher exact test to determine whether HSF-bound motifs were associated with each factor compared to HSF-free motifs and vice versa (Table S1). Depicted in Figure 4 and Figure S11 are the fractions of HSEs that are present within a given region of enrichment (enriched is colored yellow, unenriched is blue). Strikingly, only 30 (7%) inducible HSF-bound sites do not contain any tested activation marks prior to HS. This analysis reveals a statistically significant association (p-value<0.05) of HSF-bound motifs with 17 different histone modifications or chromatin-bound factors that have previously been shown to be associated with active chromatin (Table S1, Figure 4, and Figure S11), regardless of whether the motifs are classified as intergenic, promoter proximal or within genes (Table S2, Table S3, and Table S4). Unlike previous genome-wide TF binding data that show the co-occupancy of many TFs and histone marks, we are able to show that these chromatin features are present before any detectable HSF binding (Figure S12).

We have shown that the presence of activation marks strongly influences the pattern of HSF binding, so we next determined whether quantitative differences in individual marks play a role in the degree of HSF binding. For each HSE that is enriched for a mark or factor in Figure 4, we compared the ChIP-chip intensity of each mark or factor during NHS to the intensity of induced HSF binding following HS. We found a modest, but significant (p-value<0.05), correlation between the intensity of BEAF, tetra-acetylated H4, and H3K18ac with HSF binding intensity (Figure S13).

**Figure 5:**
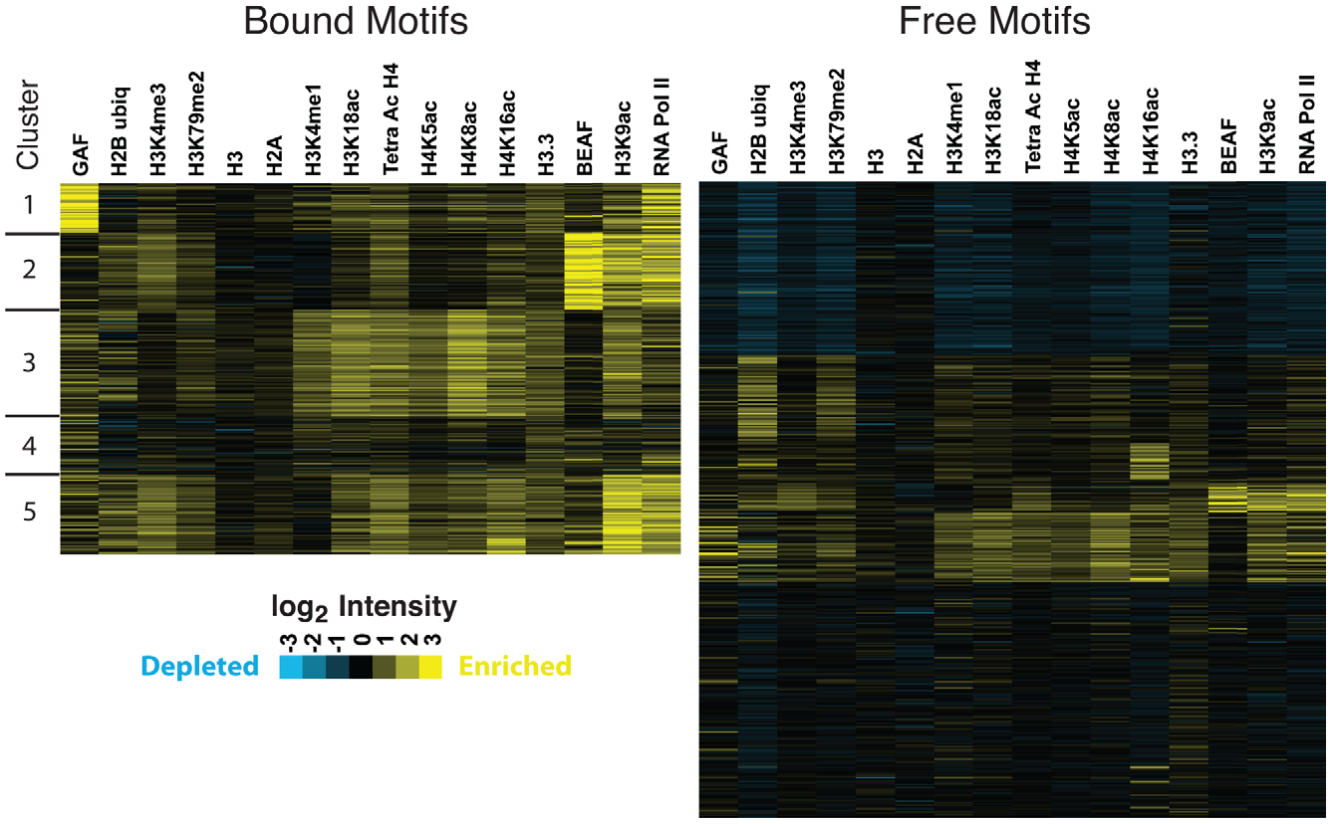
Active chromatin marks are clustered at HSE motifs. K-means clustering analysis, specifying five clusters, reveals that the histone modifications tend to occur together at HSF-bound motifs. Each motif corresponds to an individual row. Columns represent the average microarray intensity of all the probes in a 400 base window centered on the motif for a given factor or histone modification. Cluster and Treeview were used to generate and visualize the clustering data [59], [94].

Considering that the intensity of any one mark only modestly affects HSF binding, we set out to determine whether distinct patterns of TF profiles and histone modifications affect HSF binding intensity. Sets of histone modifications and TFs occur together in distinct combinations on the genome-wide scale in eukaryotic cells [18], [33], [55], [56], and this chromatin landscape can be used to predict and characterize functional regions of the genome [57], [58]. We used cluster analysis [59] to determine whether TF factors and histone modifications showed clear binding patterns at both classes of HSE motifs (Figure 5). This clustering shows that, generally, any single HSF-bound motif is enriched for many activation marks. HSF-free motifs are primarily found in regions with background levels or depleted levels of activation marks. Consistent with our composite profiles, nucleosomal H3 and H2A were not depleted at the bound HSEs prior to HSF binding and H3.3 is generally enriched. Our findings indicate that HSF-accessible chromatin is not synonymous with nucleosome vacancy, but rather, with marks of loose or active chromatin.

Clusters are not absolutely delineated by the presence or absence of a given factor or set of factors; however, we note general properties of individual clusters. For instance, ubiquitous acetylation of histone residues and high levels of H3K4me1 characterize HSF-bound cluster three, while cluster four contains modest levels of every factor and modification tested (Figure 5). Considering that motif conformity does not significantly affect HSF-signal (Figure S7A and S7B), we tested whether clustering HSEs cleanly separated strong and weak binding sites. We observe that cluster four generally exhibits less intense HSF binding, while cluster one, which is driven by intense Pol II and GAF signal, contains stronger HSF binding sites (Figure S7C). These patterns, however, are not sufficient to account for differences in HSF binding intensity, as the HSF intensity in any p-value quartile or cluster overlaps with all other classes. Ultimately, it is likely that the rules that govern TF binding and intensity of binding are a complex nonlinear system, which results in motif accessibility.

### Chromatin landscape dictates HSF binding to a target motif

**Figure 6:**
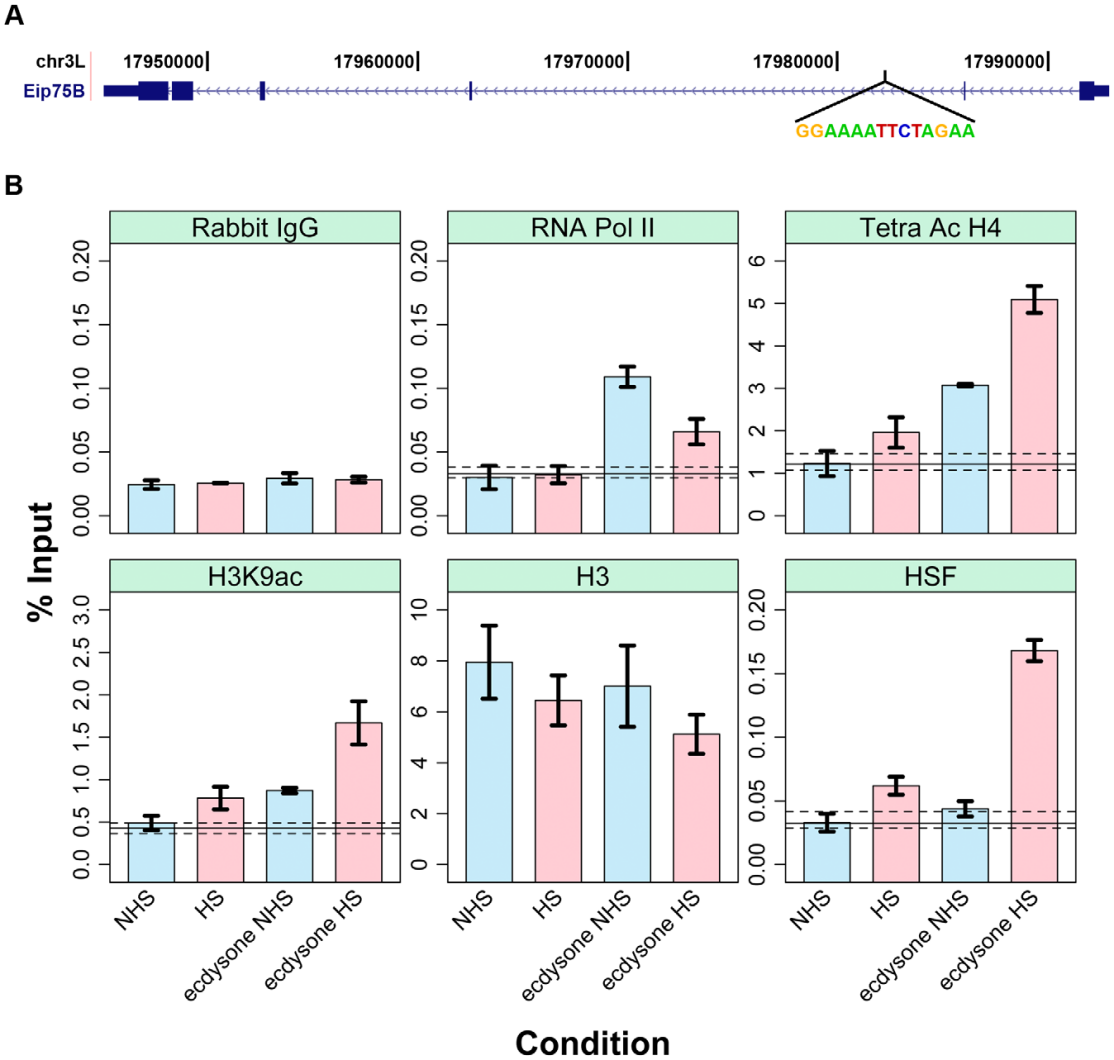
Changing the chromatin landscape converts an HSF-free motif to an HSF-bound site. (A) The ecdysone inducible gene *Eip75B* harbors an HSF motif that conforms to the consensus with a p-value of 1.2×10^−7^. (B) The blue bars represent the changes in factor and histone modification occupancy after ecdysone is added to the cells. The pink bars indicate the changes in occupancy after HS treatment in cells that were pre-treated with ecdysone. Precipitation with Rabbit IgG controls for non-specific pull-down at this site for each condition (first sub-panel) and dashed and solid lines indicate the range of background intensities for non-specific background pull-down by each antibody (see Materials and Methods) and provides an estimated threshold for assessing enrichment over background.

The strong correlation between open chromatin and HS-induced HSF suggests that open chromatin dictates HSF accessibility. To test this hypothesis, we directed a change in the chromatin landscape, from the restrictive to the permissive state, at an unbound HSE and then examined HSF binding following HS. HSF has been shown to selectively occupy the ecdysone inducible 75B cytological locus, only when the locus is transcriptionally “puffed”, in salivary gland cells [19]. We found an HSF-free motif that resides within the body of an ecdysone inducible gene isoform, *Eip75B,* which can be inducibly expressed in S2 cells (Figure 6A) [60]. We confirmed that this motif is minimally bound by HSF after HS, and is below the threshold for peak detection by ChIP-seq (Figure 6B). Ecdysone treatment alone results in RNA Pol II recruitment to the body of the *Eip75B* gene, but does not affect HSF occupancy of the HSE motif (Figure 6B). H3K9ac and tetra-acetylated H4 increase above the background threshold (top dashed line), while H3 levels are unaffected after a 30′ ecdysone treatment (Figure 6B). Recall that prior to HS, between 70% and 80% of the HSF-bound HSEs are significantly enriched for each RNA Pol II, tetra-acetylated H4 and H3K9ac (Figure 4). A 30-minute ecdysone pre-treatment changes the chromatin landscape and allows HSF to strongly occupy the motif following HS (Figure 6B).

Pre-treatment with ecdysone, followed by HS, not only allows HSF binding at this HSE, but also causes a concomitant increase in local H4 and H3K9 acetylation and decrease in RNA Pol II intensity (Figure 6B and Figure S12). Increased acetylation of histones is consistent with HSF's ability to recruit the acetyltransferase CREB Binding Protein (CBP) to HSF bound sites [61], [62]. At first glance, it is unintuitive that RNA Pol II intensity is compromised following heat shock (Figure 6B and Figure S12). However, this molecular analysis confirms a long-standing observation that following HS, HSF has the ability to repress ecdysone inducible puffs and general protein synthesis [19], [63]. While the mechanism of HSF-mediated repression is unknown in *Drosophila*, it is tempting to speculate that HSF can act as a roadblock to RNA Pol II within the bodies of active genes (Figure S14).

### Promoter-bound HSF does not necessarily lead to gene activation

**Figure 7:**
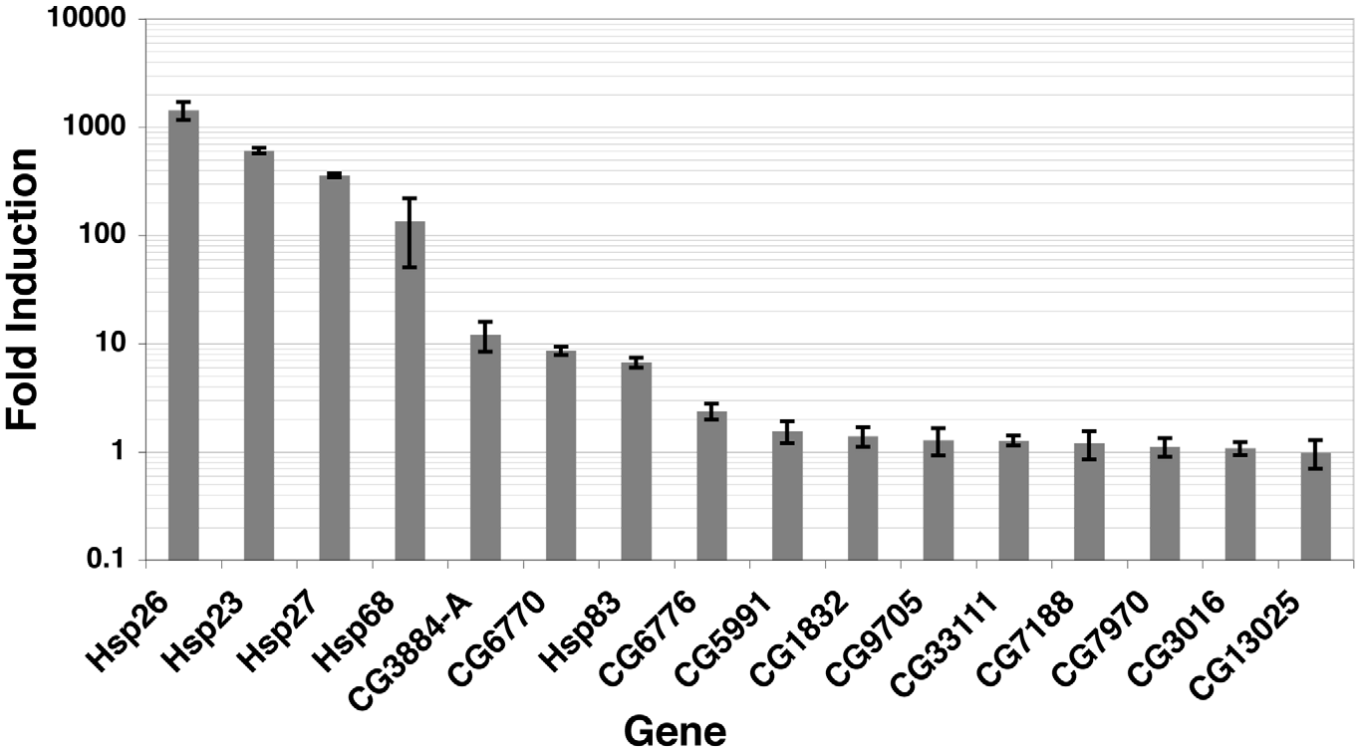
Induced mRNA accumulation after a 20′ HS shows that promoter-bound HSF has varying induction effects. Oligo dT-reverse transcribed RNA was subjected to real-time qPCR with the primers illustrated in Figure S15 (sequences available within Dataset S6), during NHS and 20′ HS conditions. All mRNA levels were normalized to *RpL32* [95] and are represented as HS mRNA levels divided by NHS levels. Three independent biological replicates and two technical replicates for each biological sample were performed.

It has long been known that HSF inducibly binds to many sites and only a subset of sites are transcriptionally activated by HS [19], [64]. These studies, however, did not have the resolution to determine if HSF binding sites did not lead to mRNA production simply because HSF was not promoter-bound. In all well-characterized cases of *Drosophila* HSF-induced transcription, HSF binds to the promoter. To determine whether promoter-bound HSF is sufficient to upregulate the local gene, we measured mRNA abundance at candidate genes during NHS and after a 20′ HS (Figure 7). Note that HSF is inducibly bound at each gene after 2′ minutes of HS (Figure S15), allowing sufficient time for mRNA accumulation (reviewed in [65]). We observe a continuum of induced mRNA accumulation, from the highly induced *Hsp26* gene, to genes that are unaffected by HSF binding (Figure 7).

Previous genome-wide ChIP experiments report that TF binding intensity generally correlates with functional binding [18], [66]. The ChIP-seq signals that we observe are directly comparable to qPCR quantified ChIP material, indicating that the quantitative properties of ChIP were retained in our sample preparation (Figure S16). Because HSF acts as a potent acidic activator [67], we hypothesized that all genes that exhibit inducible and strong promoter binding of HSF would be activated. HSF can activate when bound moderately to the promoters of genes, as is the case for the CG3884 and CG6770 genes (Figure 7 and Figure S15). Surprisingly, HSF binds inducibly and intensely to the CG3016 and CG13025 promoters (Figure S15), but these mRNA levels remain unchanged (Figure 7). Selective activation is not unique to HSF, as both ER and p53 bind the promoters of genes in a signal-dependent manner, but transcription of some local genes remains unaffected [2], [68].

To investigate how HSF may be selectively activating local genes, we used the ChIP-chip data to look for patterns of histone modification and TF binding that separates functional promoter-bound HSF sites, which can activate gene expression, from promoter-bound HSF that does not result in gene activation. We noticed that GAF was present at many up-regulated genes, and in contrast, BEAF was present at unregulated genes (Figure S17). Previous work has shown that GAF is important for the activation of HS genes [69], but our results indicate that GAF is not necessary for HSF activation (Figure S17). BEAF has been shown to function as an insulator [31], [32]; therefore, we speculate that BEAF is blocking the activation function of HSF at unregulated genes. Previous work has implicated paused polymerase as an important criterion for activation from an *Hsp70* promoter [70]. Using promoter-proximal enriched Pol II and pausing factor (NELF) data [54], however, we did not see a significant correlation between these pausing hallmarks and activation potential using these 16 genes. In the same way that chromatin signatures affect the binding of HSF to a motif in vivo, we expect that chromatin landscape and individual gene properties act together to dictate the activation potential of activator-bound genes.

## Discussion

We present an experimental approach that increases the sensitivity and power of determining TF-bound sites by ChIP-seq, and we use this approach to characterize the binding profile for HSF under both NHS and HS conditions. Our analysis revealed that HSF binding is dependent upon an underlying HSE motif, although the primary HSE sequence is not sufficient to confer HSF binding. HSF-bound HSEs were found to be associated with a chromatin landscape that harbors active marks prior to HSF binding. Lastly, we demonstrated that promoter-bound HSF is not sufficient to activate local genes.

The ChIP-seq method is used routinely to determine genome-wide factor binding profiles; however, important controls and variations in the ChIP protocol more fully exploit this approach. Our implementation of the control RNAi knockdown of HSF allowed us to eliminate the genome-wide set of false positive signals that were resistant to this knockdown, and prevented the elimination of many true positive binding sites. Another rigorous and complementary control for specificity includes performing independent ChIP experiments with multiple antiserum preparations, each of which is affinity purified with nonoverlapping antigens [18], [38]. The details of ChIP-seq chromatin preparation can also enhance peak detection [71]–[73]. Additional crosslinking agents [74] and crosslinkers that target particular types of protein/DNA interactions, such as exclusively probing direct protein/DNA interactions with UV light [1], [75], can also augment the type and quality of information obtained by the basic ChIP-seq strategy.

The non-sequence dependent specificity observed by TFs can be explained by non-mutually exclusive mechanisms: DNA binding is specifically inhibited by repressive chromatin, aided by active chromatin, or mediated by cooperative interactions with chromatin factors. Here, we report that repressive marks contribute minimally to restrict HSF binding, as only a small fraction of HSF-free motifs are associated with repressive chromatin (Figure S11). Additionally, we observe that chromatin containing background levels of active and repressive marks is unfavorable to inducible HSF binding—the default state of an in vivo HSE can be considered inaccessible. In contrast, HSF inducibly binds to sites that contain TFs and marks of active chromatin prior to HS induction. We have shown that the chromatin landscape can be modified to the permissive state and result in recognition and binding of a previously unbound HSE. This result suggests that HSF does not primarily function to bind DNA cooperatively with other factors, but simply co-occupies the same regions as other TFs, due to the accessible nature of the DNA. These results provide a framework for understanding the binding selectivity of HSF, and we look forward to mechanistic studies that solidify the rules of in vivo binding specificity.

Activators are generally thought to bind to promoters and recruit either Pol II or coactivators to produce productively elongating Pol II. HSF recruits the acetyltransferase CREB Binding Protein (CBP) and a methyltransferase, Trithorax, directly to HS genes [61], [62]. Paradoxically, this study shows that the chromatin landscape at HSF binding sites contains considerable histone acetylation and methylation prior to detectable HSF binding. HSF recruits these enzymes after HS to broaden the domain or increase the level of histone modifications (Figure 6 and Figure S12). Another, non-mutually exclusive, possibility is that cofactors other than histones are the functional targets of recruited transferases. Although we describe the landscape at HSF binding sites prior to HS, it still remains unclear which factors are responsible for setting up or maintaining the accessibility of these motifs. Furthermore, many HSF-binding sites are probably passively occupied because they happen to be accessible and HSF binding is non-deleterious [76], but these sites likely have no function in the HS response. The global chromatin landscape is dynamic throughout development and environmental changes; therefore, we expect that the HSF binding profile at non-functional sites is dynamic as well. Nonetheless, the HS response is a ubiquitous cellular response, so functional sites are likely to be evolutionarily constrained at the sequence level [77], [78], and actively maintained in the accessible state at the level of chromatin organization.

The maintenance of functional HSF binding sites may be occurring as a result of a specific class of activators. Non-traditional activators, such as GAF, are known to recruit cofactors that establish an accessible chromatin state, as opposed to directly activating transcription of the local gene (reviewed in [79]). This general mechanism has been characterized at the *phaseolin* gene in *Arabidopsis* [80] and at the *PHO5* gene in yeast (reviewed in [81]). Taken together, this suggests a step-wise process whereby a repressed site can be potentiated for activator binding and subsequently activated. Additionally, it has been shown that active marks are not simply a product of transcription, as the active marks that are associated with intergenic DNaseI hypersensitive sites and putative enhancers are not correlated with respective gene expression [33]. Our results suggest that the landscape may be marked with active histone modifications to allow binding of activators that can stimulate transcription; therefore, the presence of a modification would not be expected to correlate with gene expression if the activator has yet to bind. Further investigation of activator binding sites during non-induced conditions will determine the generality of this observation.

Our candidate gene analysis shows that HSF is not sufficient to activate local genes. Although inducibly activated genes are occupied by their cognate transcriptional activator near the TSS [4], [82]–[84], it remains unclear how the majority of activators discriminate between locally bound genes to selectively activate. Strikingly, Caudal exhibits promoter element-specific activation, specifically activating genes that contain the Downstream Promoter Element (DPE) [85]. Previously, we presented evidence that the presence of a paused polymerase facilitates activation from an *Hsp70* promoter [70], but it is unclear whether or not this is true for the majority HSF-inducible genes. Combinations of promoter features and gene properties are likely necessary for activation. One certainty, however, is that the recent emergence of genome-wide expression and binding data makes the characterization of complex regulatory mechanisms more exciting and promising than ever.

## Materials and Methods

### ChIP

The ChIP protocol has been previously described [86]. In short, S2-DRSC (lot 181A1) cells were grown in Schneider's media with 10% FBS (lot ASD29137), consistent with modENCODE experiments. Heat shocked cells were instantaneously shifted to 36.5°C by the addition of an equal volume of 48°C media to the 25°C culture. Heat shocked cells were instantaneously cooled to room-temperature and crosslinked with a final concentration of 2% paraformaldhyde for one minute; this shorter duration of crosslinking with higher concentration of paraformaldehyde was found to increase the signal-to-noise ratio. Instant cooling to room temperature and immediate crosslinking allows the heat shock and NHS samples to be crosslinked at the same efficiency and directly compared. We cannot strictly rule out the possibility that instantaneous cooling cells to room-temperature for one minute contributes to the recovery and dissociation of HSF at lower affinity sites, including the 708 HSF-free sites. However, paraformaldehyde penetrates cells quickly to effectively block further cellular changes, and HSF's DNA binding activity is only modestly affected even after a 30 minute recovery from HS [87]. Crosslinking was quenched by the addition of glycine to a final concentration of 250 mM and the extract was sonicated as previously described [86], but for three-times the duration to increase enrichment [88]. The Protein-A beads were blocked with BSA (1 mg/ml) and Polyvinylpyrrolidone (1 mg/ml) prior to the IP and freshly thawed antiserum was used for each IP, which also increased signal compared to noise.

### Illumina sample preparation

The sample preparation was previously described [89], with some modifications. Only one size selection, after adapter ligation, was performed. Thirteen cycles of PCR were performed. Quant-iT Pico Green (Invitrogen) staining was used to quantify the DNA sample. Samples were submitted to the Cornell DNA Sequencing and Genotyping Lab and run on the Illumina Genome Analyzer II.

### RNAi

RNAi-mediated HSF knockdown was performed as previously described [69]. Primer sequences are available within Dataset S6.

### Peak Calling

Sequence tags were aligned to the *Drosophila melanogaster* April 2006 release of the reference genome using MAQ [90]. We considered those tags that aligned uniquely with less than 4 mismatches. A summary of the sequencing tag counts and unique alignment counts for each condition are supplied in Table S5. The text files containing raw sequence tags and uniquely aligned tags were deposited into NCBI's Gene Expression Omnibus (GEO) [91], accession number GSE19025. Two programs [41], [42] (referred to as MACS and SPP, respectively) were independently used to call peaks with the MAQ mapped sequences for each experimental condition. The parameters we used for each program are indicated in Figure S3. The Subpeaks package was further used to dissect the few areas of broad MACS enrichment. Using SPP, we determined that we achieved saturation at this depth of sequencing. Either of two criteria was used to consider a peak RNAi-sensitive: 1) a peak coordinate was called in both the experimental and RNAi dataset and the peak is depleted in the RNAi data more than the *Hsp83* promoter depletion; 2) a peak was only called in the experimental dataset and the corresponding region of the RNAi dataset was depleted by at least 3-fold. The intensity used to calculate depletion was defined by the normalized tag count of mapped 5′ ends in the 240 base window centered on the experimental peak center coordinate. SPP and MACS were considered to have called the same peak if the SPP peak center was within the Subpeak enrichment boundary or the broader MACS enrichment boundary. The window that corresponds to the 60 bases flanking each peak center was used as input for MEME [43]. MAST and Tallymer were used in conjunction to determine the 100% mappable (for 40mer tags in the 400 bp window centered on the motif) HSF-free motifs [43], [45].

### Chromatin Landscape Data

The individual labs that generated the chromatin landscape data also validated their results. Table S6 provides the respective modENCODE ID or GEO accession number for each dataset used in this study.

### Ecdysone Treatment

*Drosophila* S2 cells were treated with 1000×20-hydroxyecdysone (20E) in 2% ethanol, at a final concentration of 1 µM for 30 minutes. ChIP was performed immediately after 30 minutes of 20E, for the NHS treated cells, or after a 20 minute HS. Two independent experimental replicates were performed for “20E/NHS” and “20E/HS”. Control cells were treated with 2% ethanol as the vehicle. Two independent control samples were performed, and the values were compared to a no treatment control. Vehicle treatment was comparable to no treatment, so we combined the measurements for a total of three independent biological replicates for both NHS and HS conditions. The error bars indicate the standard error of the mean. Importantly, we calculated two important background measurements. First, we performed ChIP with Rabbit IgG for each condition, to control for non-specific pull-down by IgG or beads. Secondly, we performed ChIP-qPCR at eight regions where each factor or modification is not enriched in untreated conditions [29], which controls for non-specific background pull-down by each antibody. Generally, the background IP by histone modification antibodies is high as measured by raw percent input, presumably do to cross reaction with unmodified histones, so this measurement is necessary in order to assign a threshold for enrichment in ChIP-qPCR assays (the top dashed line).

### mRNA Reverse Transcription

RNA levels were measured as previously described [92].

### Real-Time qPCR

Dataset S6 contains the primer sets that were used for measuring mRNA abundance. Table S7 contains the primer sequences that were used for ChIP-qPCR.

## Supporting Information

Figure S1 Experimental replicates correlate as measured by Pearson correlation coefficient. The normalized density of tag counts for each peak coordinate is calculated by dividing the tag counts in the 300 base window centered on the HS peak summit by the sum of the tag counts in all peak windows. The density values were plotted against one another and the Pearson coefficient was calculated. Note that the *Hsp83* promoter has the highest density of tag counts during NHS.
(0.63 MB TIF)

Figure S2 UCSC Genome Browser screen shot of the *Hsp83* gene, which harbors the highest affinity HSF binding site. The HSF intensity at the *Hsp83* promoter of HSF-depleted cells decreases to less than 70% of untreated cells, for both NHS and 20′ HS conditions. The y-axis scale is linear (from 3 to 350) and directly comparable between all plots (shifted tags/10bp/10 million sequences in the library).
(1.54 MB TIF)

Figure S3 Peak calling workflow. Two peak calling programs were used to call peaks with relaxed parameters. All peaks were filtered based on their sensitivity to HSF-RNAi depletion. Without the HSF-RNAi control data, we would have obtained 310 RNAi-resistant false positive peaks and discarded 118 RNAi-sensitive lower confidence peaks. SPP and MACS called 333 of the same peaks and 131 peaks were unique to either program.
(1.09 MB TIF)

Figure S4 HSE distribution relative to peaks. We determined the distance (in bases) between the program-called peaks, or a randomly chosen euchromatic reference genome coordinate, and the closest HSE with a p-value below 0.001. The probability distribution function for each panel is colored red; the cumulative distribution function for each panel is shaded grey. More than 85% (400/464) of the RNAi-sensitive peaks have a motif within 20 bases; more than 95% (442/464) of the RNAi-sensitive peaks have a motif within 60 bases. In contrast, the distribution of HSEs relative to RNAi-resistant peaks mirrors the distribution of motifs relative to randomly chosen regions.
(2.18 MB TIF)

Figure S5 Inducible binding of HSF to *Hsp70*. In *Drosophila* S2 cells there are five copies of the most well-characterized HS responsive gene, *Hsp70* (Gilmour and Lis, 1986). Due to complications in mapping sequence reads uniquely at these genes, we show the average intensity of HSF binding on a composite *Hsp70* gene. The y-axis scale is normalized to the six *Hsp70* genes in the reference genome and directly comparable between all plots (shifted tags/10bp/10 million total, uniquely and non-uniquely alignable, sequences in the library). The conspicuous peak seen in the preimmune-IP corresponds to the paused RNA Pol II of *Hsp70*; this peak is likely the result of a residual strong Sono-seq peak, which are found to co-associate with Pol II [73]. Gilmour DS, Lis JT. (1986) RNA polymerase II interacts with the promoter region of the noninduced *Hsp70* gene in *Drosophila melanogaster* cells. Mol Cell Biol 6: 3984-3989.
(1.62 MB TIF)

Figure S6 The majority of motifs occupied by HSF during HS are not sensitive to HSF-depletion during NHS conditions. For the NHS condition, we compared the ratio of experimental signal to HSF-KD signal (designated “Ex/KD”) at the 422 HS peaks that were not detected under NHS conditions. Signal is defined as the normalized tag count of mapped 5′ ends in the 240 base window centered on the motif (or random) coordinate. The distribution of Ex/KD ratios for these 422 regions under NHS is similar to the NHS Ex/KD ratio for random regions; however, there is a noticeable shoulder and slight positive shift in the distribution indicating that a fraction of the HSF-bound sites contain extremely low signal that is not sufficient to call peaks, but it is still somewhat sensitive to HSF-depletion. Note that 82% of the NHS HSF-bound ratios are found below the 5% upper tail of the random distribution, indicating that the vast majority of the signal at these sites is not sensitive to HSF depletion and thus the signal observed is likely background. In contrast, 0.5% of the HSF-bound peaks found during HS fall below the 5% upper tail of the random distribution. We anticipated that HSF is weakly bound to a fraction these sites during NHS, as the monomeric version of HSF has a double-digit nanomolar dissociation constant for a single NGAAN DNA sequence (Kim et al., 1994); likewise, a small fraction of HSF is likely trimerized and bound to full HSE motifs, as the dissociation constant for trimer-to-monomer separation of HSF is on the order of double-digit micromolar (Zhong et al., 1998; Zhong et al., 1999). The Ex/KD ratio plot for the 708 HSF-free motifs is also shown for HS and NHS conditions. We compared the Ex/KD signal ratios for the NHS and HS conditions at these HSF-free motifs to the ratios at random regions. The NHS distribution of Ex/KD ratios for these 708 motifs is similar to the HS Ex/KD ratio for random regions, but the HS distribution is slightly shifted to higher ratios and a shoulder is observed, suggesting that a small fraction of undetectable HS peaks may be weakly bound by HSF following HS. Kim SJ, Tsukiyama T, Lewis MS, Wu C. (1994) Interaction of the DNA-binding domain of *Drosophila* heat shock factor with its cognate DNA site: A thermodynamic analysis using analytical ultracentrifugation. Protein Sci 3: 1040-1051. Zhong M, Orosz A, Wu C. (1998) Direct sensing of heat and oxidation by *Drosophila* heat shock transcription factor. Mol Cell 2: 101-108. Zhong M, Kim SJ, Wu C. (1999) Sensitivity of *Drosophila* heat shock transcription factor to low pH. J Biol Chem 274: 3135-3140.
(0.91 MB TIF)

Figure S7 Distinct sets of bound HSEs have modest effects on HSF binding intensity. (A) We separated bound HSEs based on their p-value for the 20′ HS samples; then we counted sequence tags in the 240 base window centered on the motif, normalizing for the number of motifs in the window. The top two quartiles, which have the most significant p-values, generally have more tag counts than the less significant quartiles. (B) The composite HSE motif for each p-value quartile is illustrated using WebLogos [93]. (C) Clustered sets of HSF-bound motifs (Figure 5) were analyzed in the same manner as panel (A).
(1.41 MB TIF)

Figure S8 The distribution of unbound HSEs mirrors sequence annotation classes. (A) The 708 HSF-free motifs (Dataset S4) are found within gene annotations (446) and promoters (39). There are 13 unbound HSEs that are present *both* within a promoter and a RefGene body. The composite HSE for all 708 HSF-free motifs is illustrated using WebLogos [93]. (B) The sequence annotation class composition of the reference genome.
(1.22 MB TIF)

Figure S9 ModENCODE ChIP-chip signals are directly comparable to NHS ChIP-qPCR signals. ModENCODE ChIP-chip signals were plotted against ChIP-qPCR intensities for both NHS (A) and HS (B) conditions. These plots confirm that the modENCODE experiments were performed under unstressed conditions that are comparable to our experiments. These plots in panel (A) show that the intensities of the modifications are correlated between modENCODE and our NHS data at 10 HS-inducible HSEs. We also performed the same correlation test with HS cells and we see considerably decreased correlations, consistent with HSF's ability to repress transcription genome wide [19], [63] and recruit the acetyltransferase CBP, which primarily acetylates H4 (Ludlam et al., 2002). The Pearson correlation coefficient is indicated in the top-left of each panel. Ludlam WH, Taylor MH, Tanner KG, Denu JM, Goodman RH, et al. (2002) The acetyltransferase activity of CBP is required for wingless activation and H4 acetylation in *Drosophila melanogaster*. Mol Cell Biol 22: 3832-3841.
(1.24 MB TIF)

Figure S10 Factor occupancy around HSE motifs. The average factor occupancy in 100 base windows (step size of 50) around HSF-free HSE motifs (red) and HSF-bound HSE motifs (green). HSF-bound motifs are categorized by annotation class: motifs within promoters (magenta), RefGene bodies (blue), and intergenic regions (black). Enrichment at HSF-bound motifs is depicted by pastel purple, all others are colored orange.
(1.67 MB TIF)

Figure S11 HSF-bound HSE motifs are associated with marks of active chromatin. The fraction of both bound and unbound HSEs in regions of significant enrichment for a given factor or histone modification. Fisher's exact test was used to determine the association between either HSF-bound or HSF-free motifs and each modENCODE factor or histone modification (Table S1). The yellow fraction of the bar chart represents HSF binding sites that are in regions of significant enrichment, while blue depicts all non-enriched sites. A small fraction of HSF-free motifs are statistically associated with marks of repressed chromatin (Polycomb, H3K9me2, H3K9me3, H3K27me3, and H3K36me3).
(1.28 MB TIF)

Figure S12 H3K9 acetylation, tetra-acetylated H4 and RNA Pol II are enriched at inducibly bound HSEs in our NHS cell populations. (A) We performed ChIP-qPCR for RNA Pol II, H3K9ac, H4TetraAc *and* HSF in cells for a subset of HSF-binding sites (Figure S10) that were shown to contain RNA Pol II, tetra-acetylated H4 and H3K9ac in modENCODE experiments. Under NHS conditions, HSF is undetectably bound, but the activation marks are present. The blue and pink bars represent NHS and 20′ HS occupancy, respectively, for each factor. Precipitation with Rabbit IgG controls for non-specific pull-down at this site for each condition (first sub-panel) and dashed and solid lines indicate the range of background intensities for non-specific background pull-down by each antibody (see Materials and Methods) and provides an estimated threshold for assessing enrichment over background. Taken with Figure S10, we conclude that the modENCODE conditions and our conditions are directly comparable. (B) Enlargement of Tetra Ac H4 and H3K9ac plots in panel (A).
(2.94 MB TIF)

Figure S13 The intensity of activation marks prior to HS modestly correlates with induced HSF binding levels. At those sites that were enriched for a given mark or factor in Figure 4, the modENCODE ChIP-chip intensities for each HSE were correlated to the HSF binding intensity after HS. ChIP-chip intensity is defined as the average microarray intensity of all the probes in a 400 base window centered on the motif. HSF binding intensity is defined as the number of tags whose 5′ ends map in the 240 base window centered on the HSE. The Pearson correlation coefficient is indicated in the top-left of the panel. Only BEAF, tetra-acetylated H4 and H3K18ac had significant correlations with p-values below 0.05.
(1.26 MB TIF)

Figure S14 HSF may act as a roadblock to RNA Pol II. We perform RNA Pol II ChIP-qPCR at sites flanking the HSE shown in Figure 6. The widths of the bars span the genomic coordinates of the qPCR amplicons. After ecdysone treatment, there are comparable amounts of RNA Pol II at each site; however, there is a modest depletion of RNA Pol II downstream of the HSE following HS. This finding lends support a model whereby HSF can act as a roadblock to repress transcription.
(7.02 MB TIF)

Figure S15 Promoter-bound HSF has varying induction effects. Each gene from Figure 7 is inducibly bound by HSF as early as 2 minutes after heat shock (the top track of each panel). The RT-qPCR assay in Figure 7 was performed after 20 minutes of HS, which allows 18 minutes for mRNA accumulation. The qPCR primers for assaying mRNA levels are illustrated above the gene annotation.
(3.89 MB TIF)

Figure S16 ChIP-seq quantification recapitulates ChIP-qPCR intensities. The ChIP-seq peaks at 25 loci, representing a wide range of intensities, were quantified by normalizing the tag count density in the 320 base window centered on the HSE motif. The corresponding region was quantified by qPCR, using IPed DNA from an independent biological replicate that did not undergo size selection or amplification. The primers for qPCR and the intensities for ChIP-seq and ChIP-qPCR are listed in Table S7.
(7.57 MB TIF)

Figure S17 Chromatin landscape at HSF-bound promoters. Each HSF-bound promoter corresponds to an individual row. Rows are arranged from top to bottom by decreasing fold-induction after HS (Figure 7). Columns represent the average microarray intensity of all the probes in a 400 base window centered on the motif for a given factor or histone modification.
(2.24 MB TIF)

Table S1 Fisher statistic comparing the association of all HSF-bound and all HSF-free motifs with each modENCODE factor.
(0.04 MB XLS)

Table S2 Fisher statistic for promoter associated motifs.
(0.04 MB XLS)

Table S3 Fisher statistic for motifs within annotated genes.
(0.04 MB XLS)

Table S4 Fisher statistic for intergenic motifs.
(0.04 MB XLS)

Table S5 Sequence tag counts and unique alignment counts for each condition.
(0.03 MB XLS)

Table S6 ModENCODE identification number or GEO accession number for each modENCODE dataset used.
(0.04 MB XLS)

Table S7 Primer sequences for ChIP-qPCR and the raw intensity values for ChIP-seq and ChIP-qPCR.
(0.04 MB XLS)

Dataset S1 464 program called, RNAi-sensitive HSF peaks.
(0.04 MB TXT)

Dataset S2 442 HSE motifs found underlying the 464 peaks.
(0.02 MB TXT)

Dataset S3 97 HSF-bound motifs found 500bp upstream of annotated TSSs.
(0.00 MB TXT)

Dataset S4 708 HSF-free motifs.
(0.03 MB TXT)

Dataset S5 422 HSF-bound motifs whereby HSF is not detected under NHS conditions.
(0.02 MB TXT)

Dataset S6 Primer sets for HSF-RNAi and mRNA quantification.
(0.00 MB TDS)
