# Supplementary figures and images for: Chromatin Landscape Dictates HSF Binding to Target DNA Elements
Source: PLoS Genet. 2010 Sep 9;6(9):e1001114. doi: 10.1371/journal.pgen.1001114 (PMC2936546; doi:10.1371/journal.pgen.1001114)

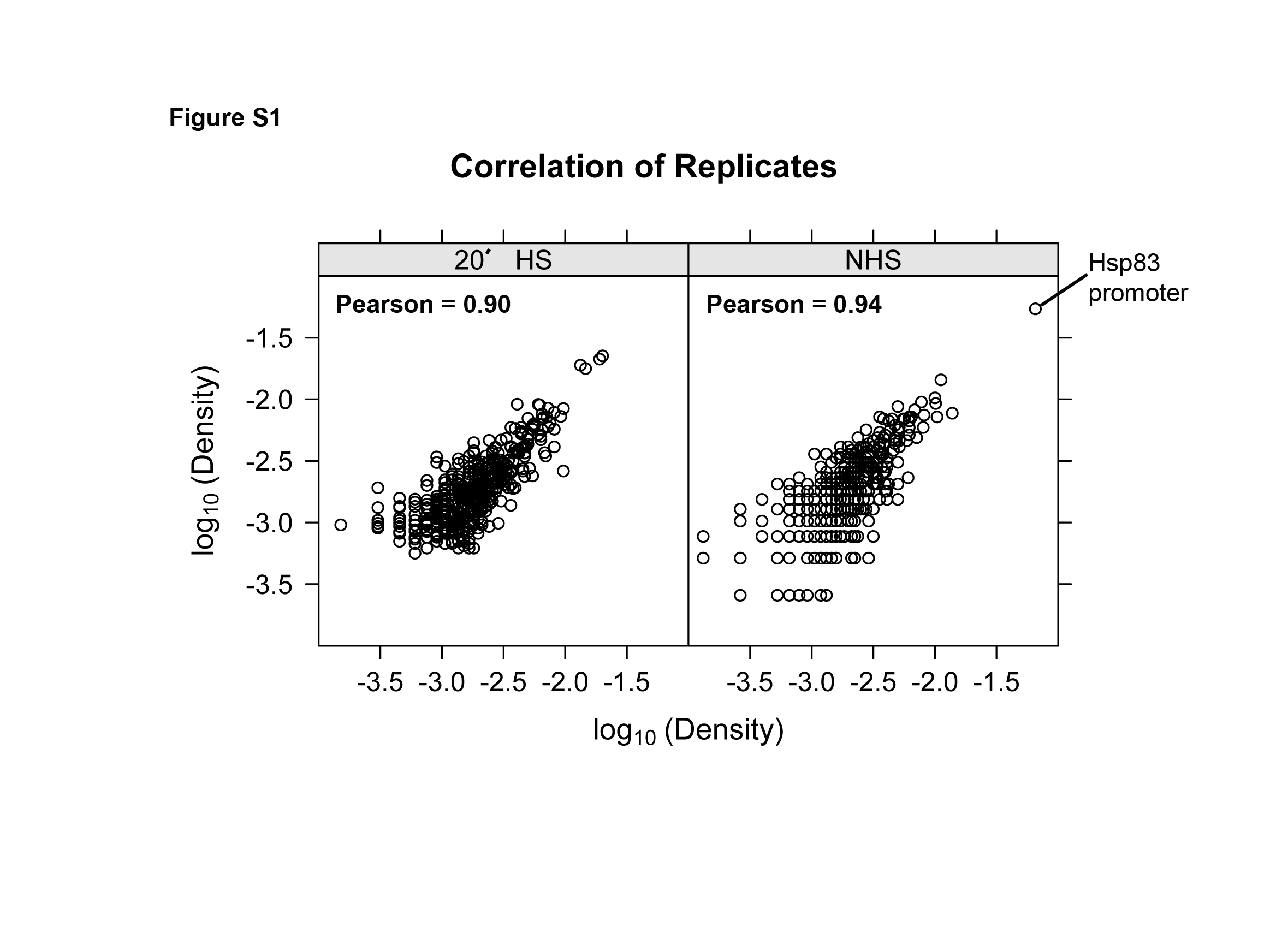

Supplement: Figure S1 — Experimental replicates correlate as measured by Pearson correlation coefficient. The normalized density of tag counts for each peak coordinate is calculated by dividing the tag counts in the 300 base window centered on the HS peak summit by the sum of the tag counts in all peak windows. The density values were plotted against one another and the Pearson coefficient was calculated. Note that the Hsp83 promoter has the highest density of tag counts during NHS. (0.63 MB TIF) [file pgen.1001114.s001.tif]

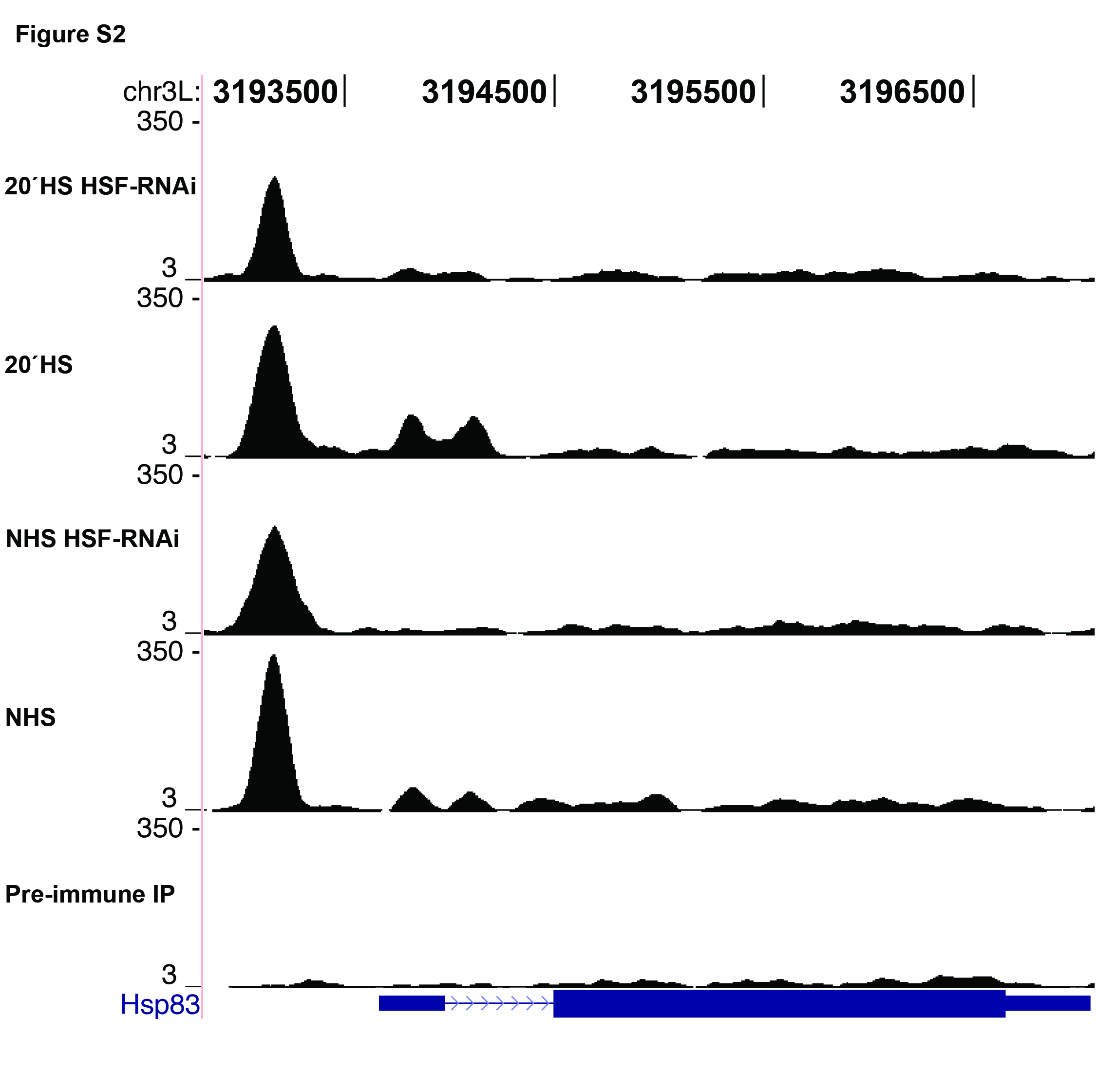

Supplement: Figure S2 — UCSC Genome Browser screen shot of the Hsp83 gene, which harbors the highest affinity HSF binding site. The HSF intensity at the Hsp83 promoter of HSF-depleted cells decreases to less than 70% of untreated cells, for both NHS and 20′ HS conditions. The y-axis scale is linear (from 3 to 350) and directly comparable between all plots (shifted tags/10bp/10 million sequences in the library). (1.54 MB TIF) [file pgen.1001114.s002.tif]

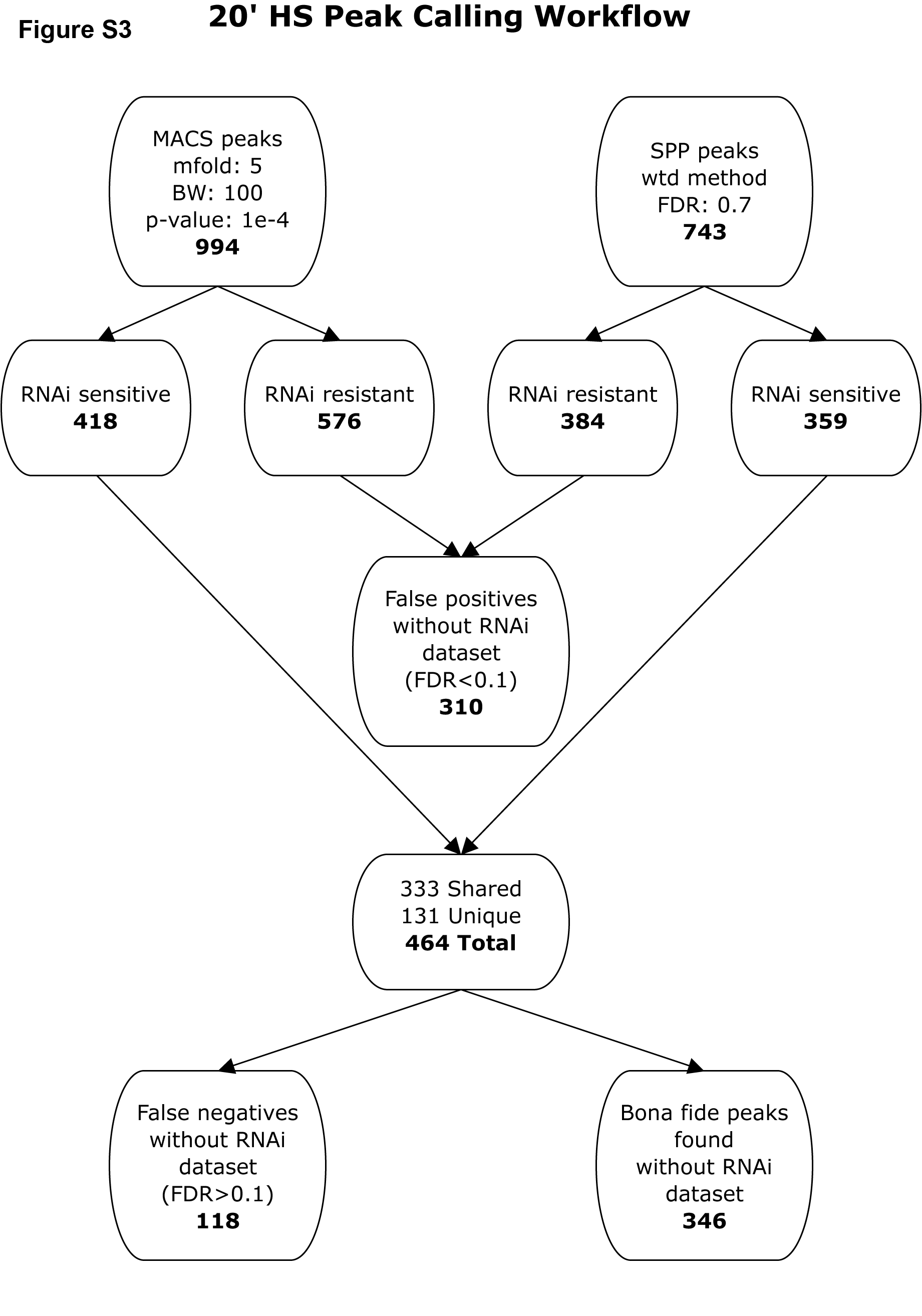

Supplement: Figure S3 — Peak calling workflow. Two peak calling programs were used to call peaks with relaxed parameters. All peaks were filtered based on their sensitivity to HSF-RNAi depletion. Without the HSF-RNAi control data, we would have obtained 310 RNAi-resistant false positive peaks and discarded 118 RNAi-sensitive lower confidence peaks. SPP and MACS called 333 of the same peaks and 131 peaks were unique to either program. (1.09 MB TIF) [file pgen.1001114.s003.tif]

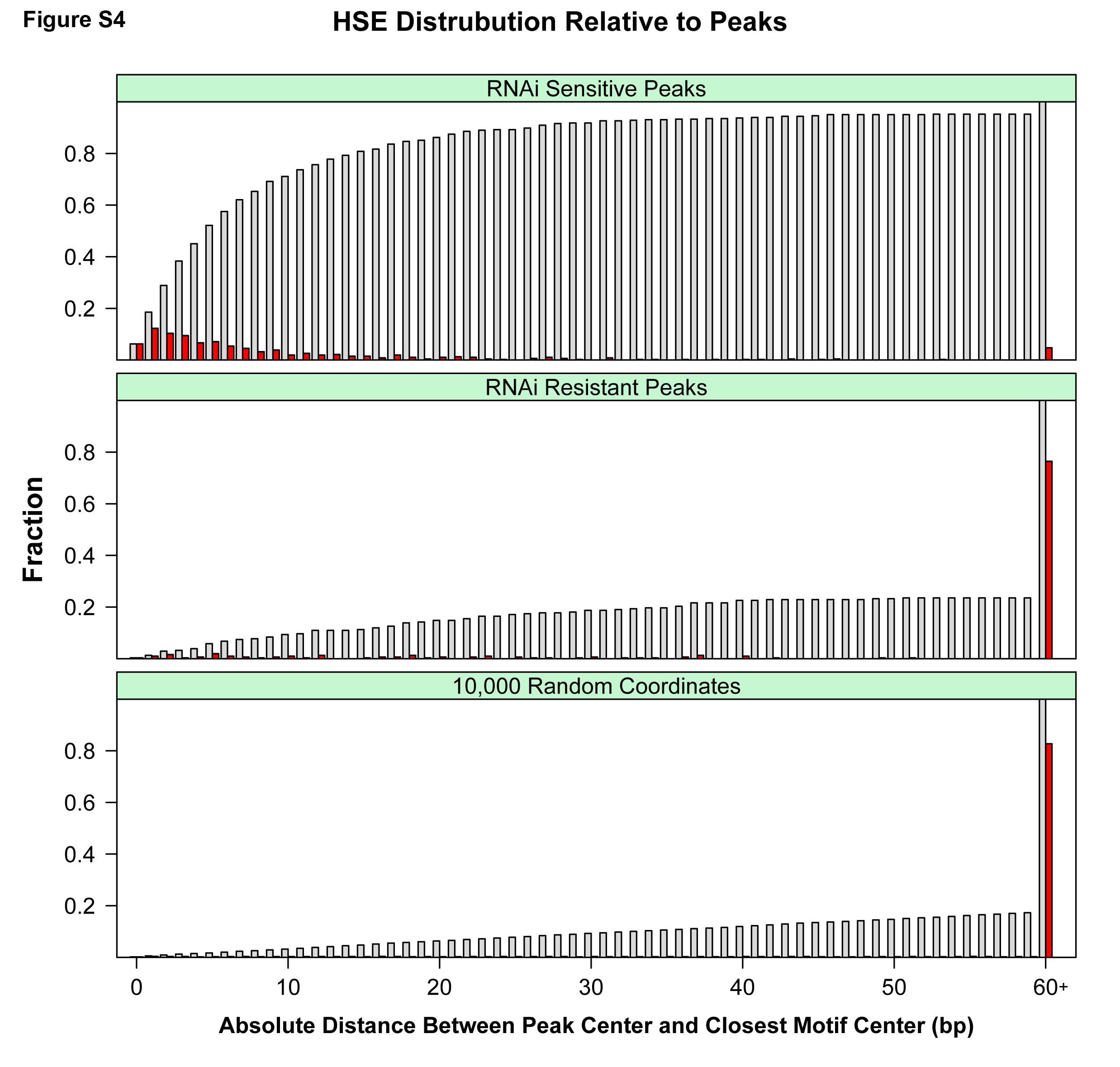

Supplement: Figure S4 — HSE distribution relative to peaks. We determined the distance (in bases) between the program-called peaks, or a randomly chosen euchromatic reference genome coordinate, and the closest HSE with a p-value below 0.001. The probability distribution function for each panel is colored red; the cumulative distribution function for each panel is shaded grey. More than 85% (400/464) of the RNAi-sensitive peaks have a motif within 20 bases; more than 95% (442/464) of the RNAi-sensitive peaks have a motif within 60 bases. In contrast, the distribution of HSEs relative to RNAi-resistant peaks mirrors the distribution of motifs relative to randomly chosen regions. (2.18 MB TIF) [file pgen.1001114.s004.tif]

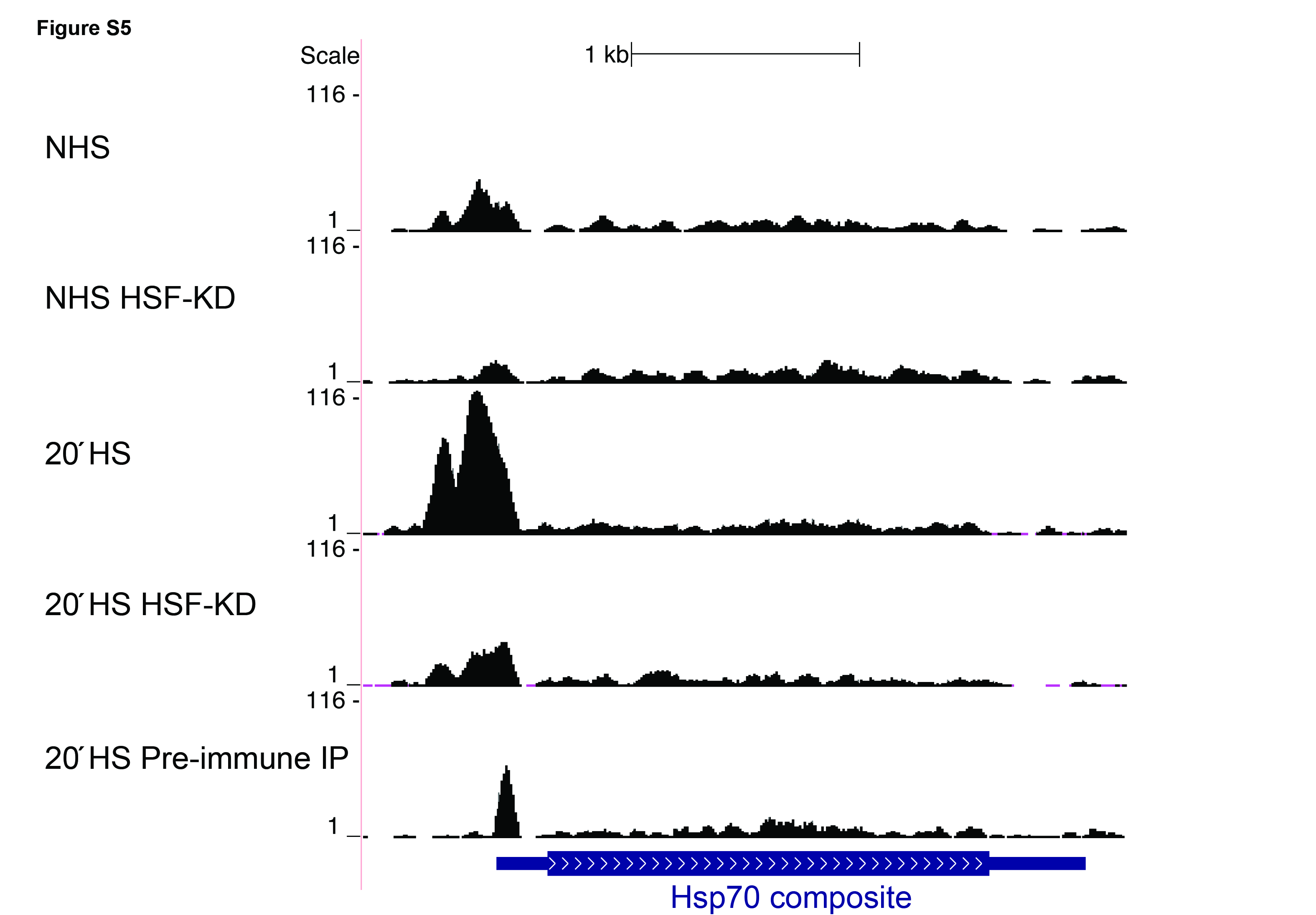

Supplement: Figure S5 — Inducible binding of HSF to Hsp70. In Drosophila S2 cells there are five copies of the most well-characterized HS responsive gene, Hsp70 (Gilmour and Lis, 1986). Due to complications in mapping sequence reads uniquely at these genes, we show the average intensity of HSF binding on a composite Hsp70 gene. The y-axis scale is normalized to the six Hsp70 genes in the reference genome and directly comparable between all plots (shifted tags/10bp/10 million total, uniquely and non-uniquely alignable, sequences in the library). The conspicuous peak seen in the preimmune-IP corresponds to the paused RNA Pol II of Hsp70; this peak is likely the result of a residual strong Sono-seq peak, which are found to co-associate with Pol II [73]. Gilmour DS, Lis JT. (1986) RNA polymerase II interacts with the promoter region of the noninduced Hsp70 gene in Drosophila melanogaster cells. Mol Cell Biol 6: 3984-3989. (1.62 MB TIF) [file pgen.1001114.s005.tif]

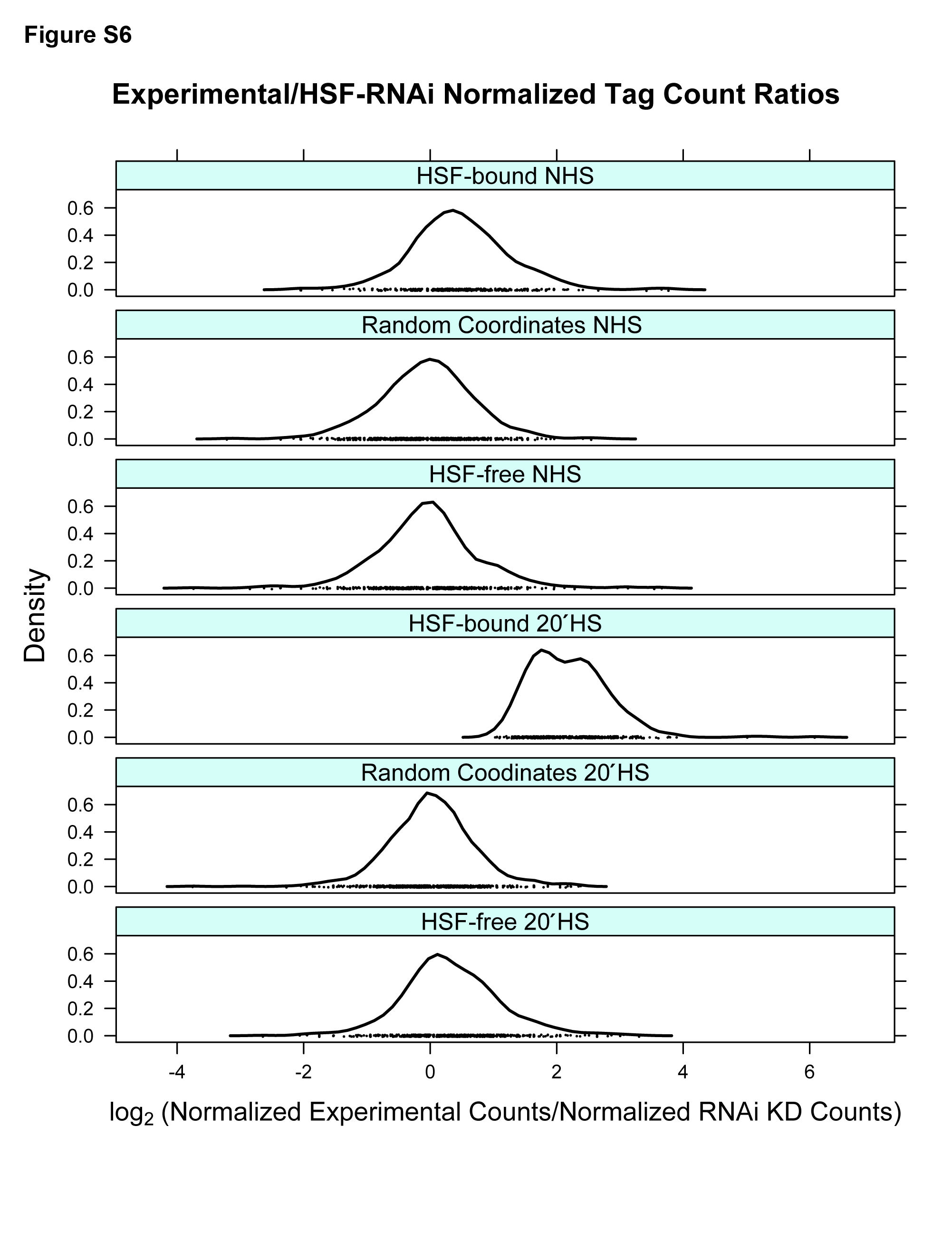

Supplement: Figure S6 — The majority of motifs occupied by HSF during HS are not sensitive to HSF-depletion during NHS conditions. For the NHS condition, we compared the ratio of experimental signal to HSF-KD signal (designated “Ex/KD”) at the 422 HS peaks that were not detected under NHS conditions. Signal is defined as the normalized tag count of mapped 5′ ends in the 240 base window centered on the motif (or random) coordinate. The distribution of Ex/KD ratios for these 422 regions under NHS is similar to the NHS Ex/KD ratio for random regions; however, there is a noticeable shoulder and slight positive shift in the distribution indicating that a fraction of the HSF-bound sites contain extremely low signal that is not sufficient to call peaks, but it is still somewhat sensitive to HSF-depletion. Note that 82% of the NHS HSF-bound ratios are found below the 5% upper tail of the random distribution, indicating that the vast majority of the signal at these sites is not sensitive to HSF depletion and thus the signal observed is likely background. In contrast, 0.5% of the HSF-bound peaks found during HS fall below the 5% upper tail of the random distribution. We anticipated that HSF is weakly bound to a fraction these sites during NHS, as the monomeric version of HSF has a double-digit nanomolar dissociation constant for a single NGAAN DNA sequence (Kim et al., 1994); likewise, a small fraction of HSF is likely trimerized and bound to full HSE motifs, as the dissociation constant for trimer-to-monomer separation of HSF is on the order of double-digit micromolar (Zhong et al., 1998; Zhong et al., 1999). The Ex/KD ratio plot for the 708 HSF-free motifs is also shown for HS and NHS conditions. We compared the Ex/KD signal ratios for the NHS and HS conditions at these HSF-free motifs to the ratios at random regions. The NHS distribution of Ex/KD ratios for these 708 motifs is similar to the HS Ex/KD ratio for random regions, but the HS distribution is slightly shifted to higher ratios and a shou [file pgen.1001114.s006.tif]

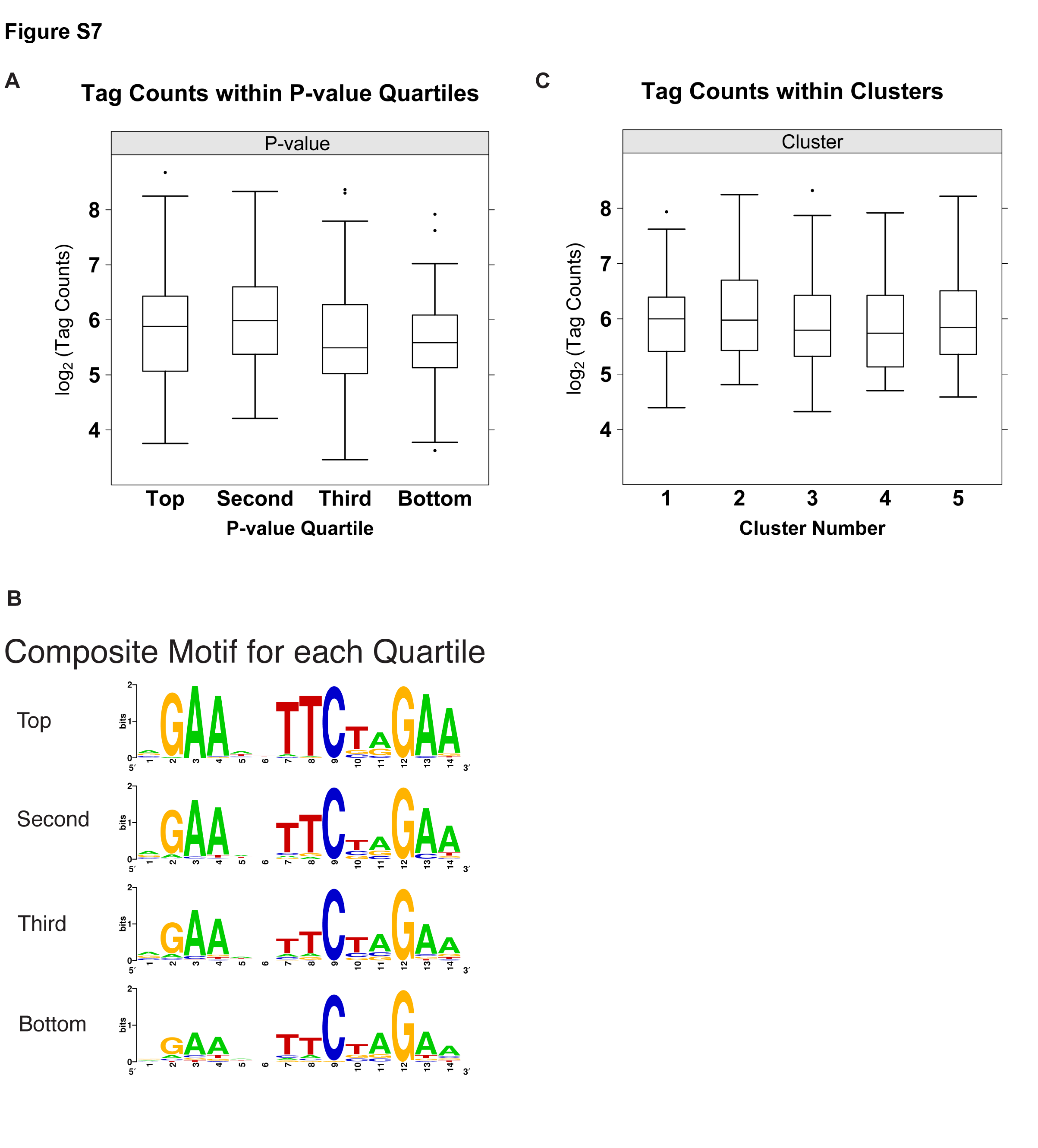

Supplement: Figure S7 — Distinct sets of bound HSEs have modest effects on HSF binding intensity. (A) We separated bound HSEs based on their p-value for the 20′ HS samples; then we counted sequence tags in the 240 base window centered on the motif, normalizing for the number of motifs in the window. The top two quartiles, which have the most significant p-values, generally have more tag counts than the less significant quartiles. (B) The composite HSE motif for each p-value quartile is illustrated using WebLogos [93]. (C) Clustered sets of HSF-bound motifs (Figure 5) were analyzed in the same manner as panel (A). (1.41 MB TIF) [file pgen.1001114.s007.tif]

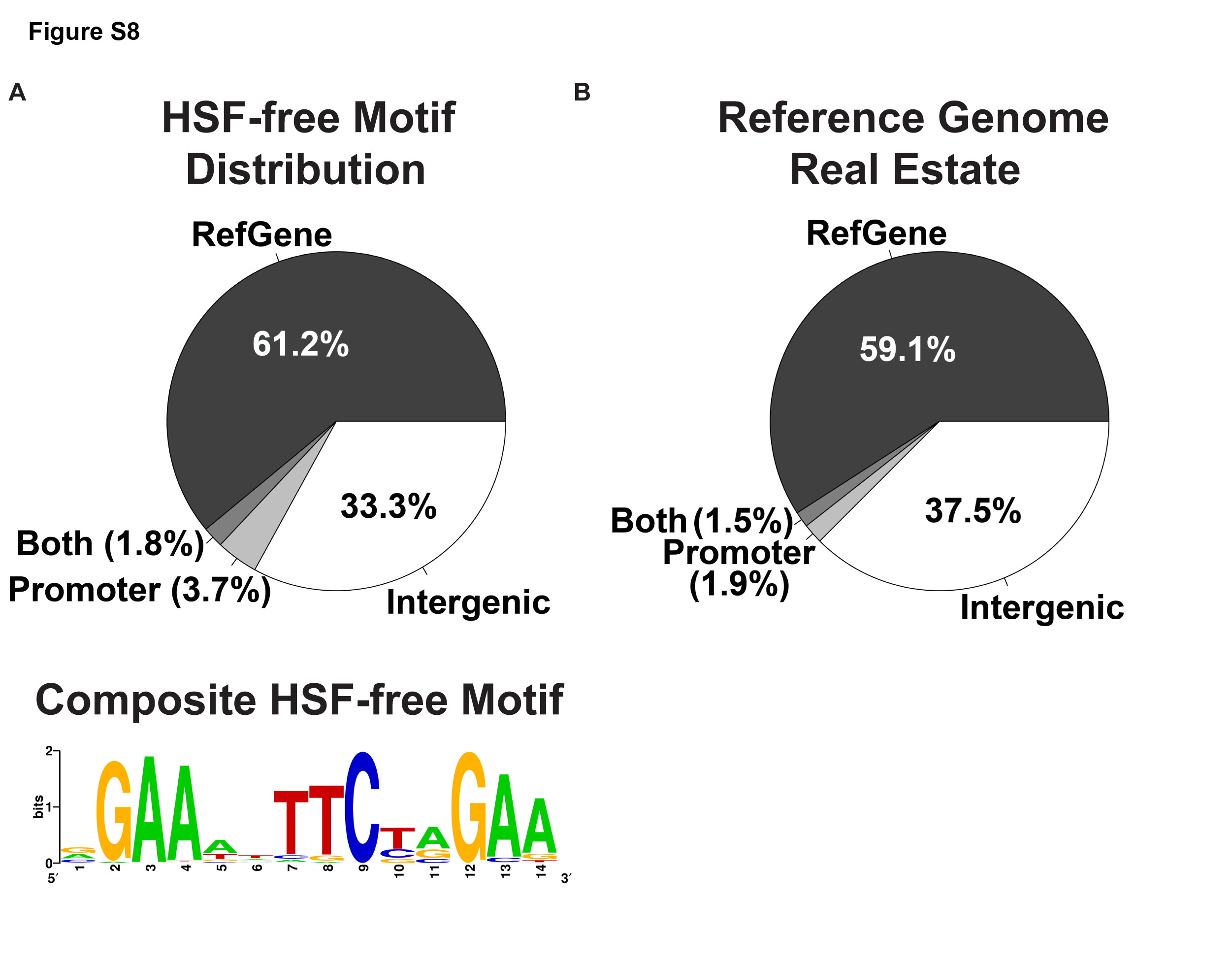

Supplement: Figure S8 — The distribution of unbound HSEs mirrors sequence annotation classes. (A) The 708 HSF-free motifs (Dataset S4) are found within gene annotations (446) and promoters (39). There are 13 unbound HSEs that are present both within a promoter and a RefGene body. The composite HSE for all 708 HSF-free motifs is illustrated using WebLogos [93]. (B) The sequence annotation class composition of the reference genome. (1.22 MB TIF) [file pgen.1001114.s008.tif]

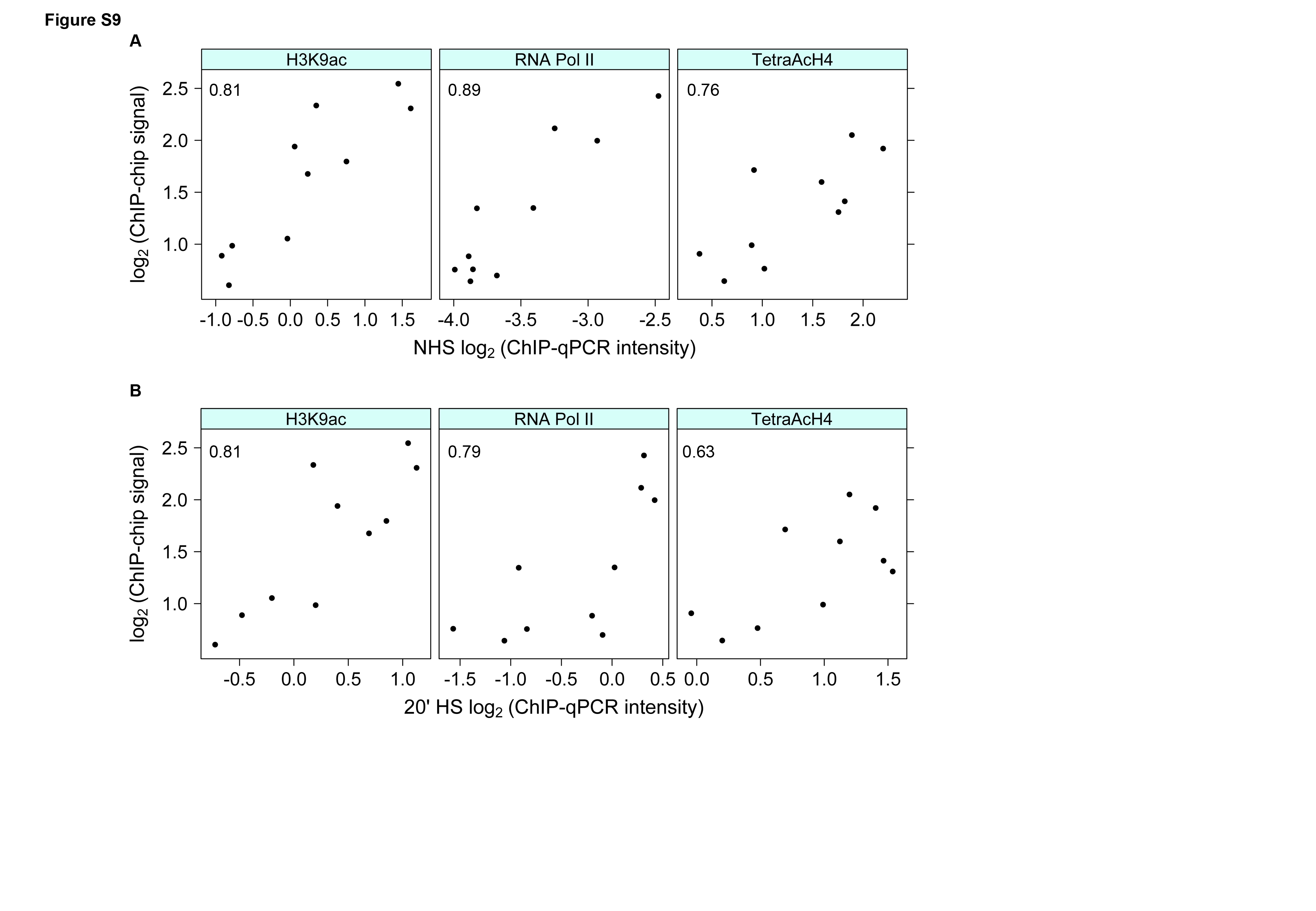

Supplement: Figure S9 — ModENCODE ChIP-chip signals are directly comparable to NHS ChIP-qPCR signals. ModENCODE ChIP-chip signals were plotted against ChIP-qPCR intensities for both NHS (A) and HS (B) conditions. These plots confirm that the modENCODE experiments were performed under unstressed conditions that are comparable to our experiments. These plots in panel (A) show that the intensities of the modifications are correlated between modENCODE and our NHS data at 10 HS-inducible HSEs. We also performed the same correlation test with HS cells and we see considerably decreased correlations, consistent with HSF's ability to repress transcription genome wide [19], [63] and recruit the acetyltransferase CBP, which primarily acetylates H4 (Ludlam et al., 2002). The Pearson correlation coefficient is indicated in the top-left of each panel. Ludlam WH, Taylor MH, Tanner KG, Denu JM, Goodman RH, et al. (2002) The acetyltransferase activity of CBP is required for wingless activation and H4 acetylation in Drosophila melanogaster. Mol Cell Biol 22: 3832-3841. (1.24 MB TIF) [file pgen.1001114.s009.tif]

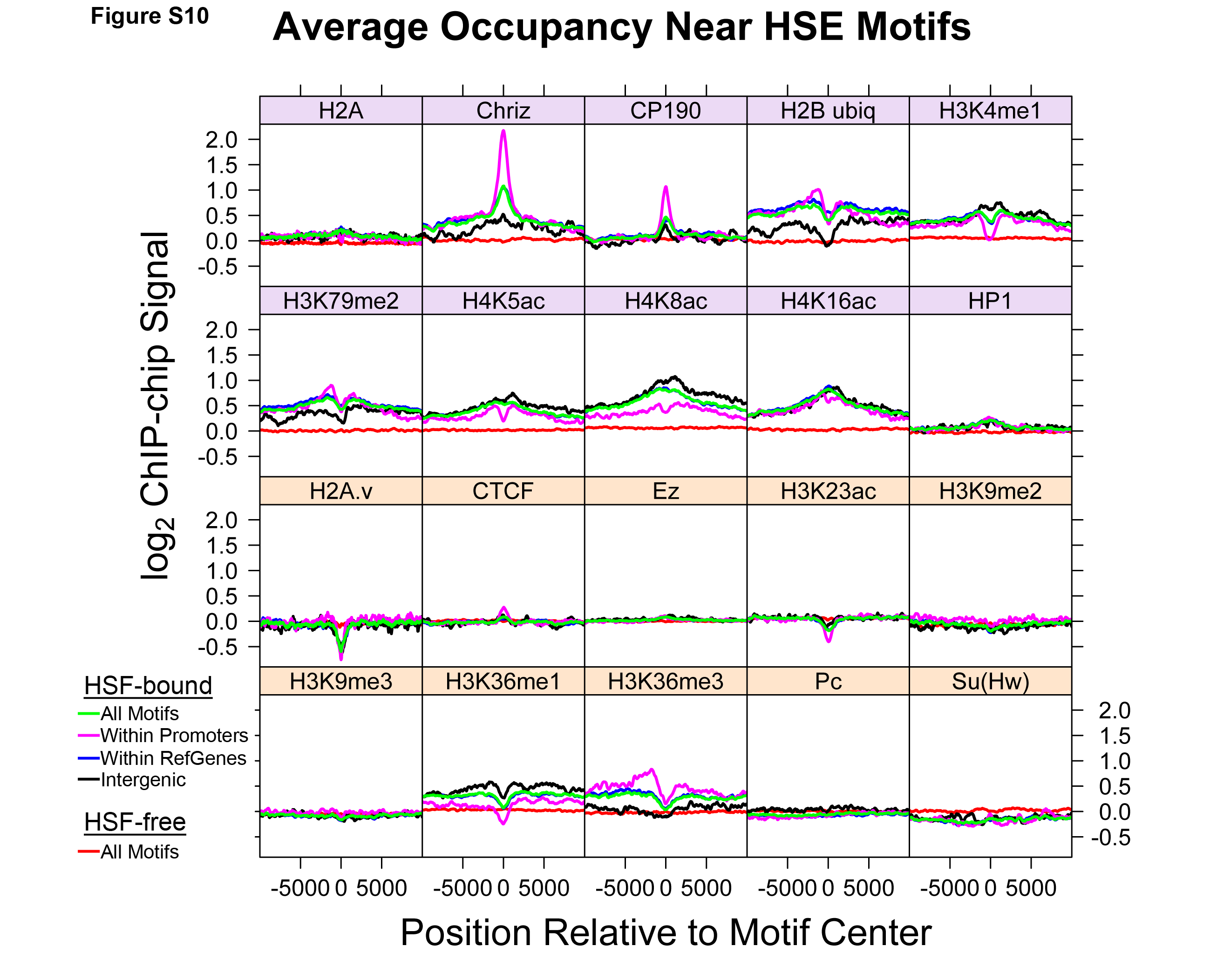

Supplement: Figure S10 — Factor occupancy around HSE motifs. The average factor occupancy in 100 base windows (step size of 50) around HSF-free HSE motifs (red) and HSF-bound HSE motifs (green). HSF-bound motifs are categorized by annotation class: motifs within promoters (magenta), RefGene bodies (blue), and intergenic regions (black). Enrichment at HSF-bound motifs is depicted by pastel purple, all others are colored orange. (1.67 MB TIF) [file pgen.1001114.s010.tif]

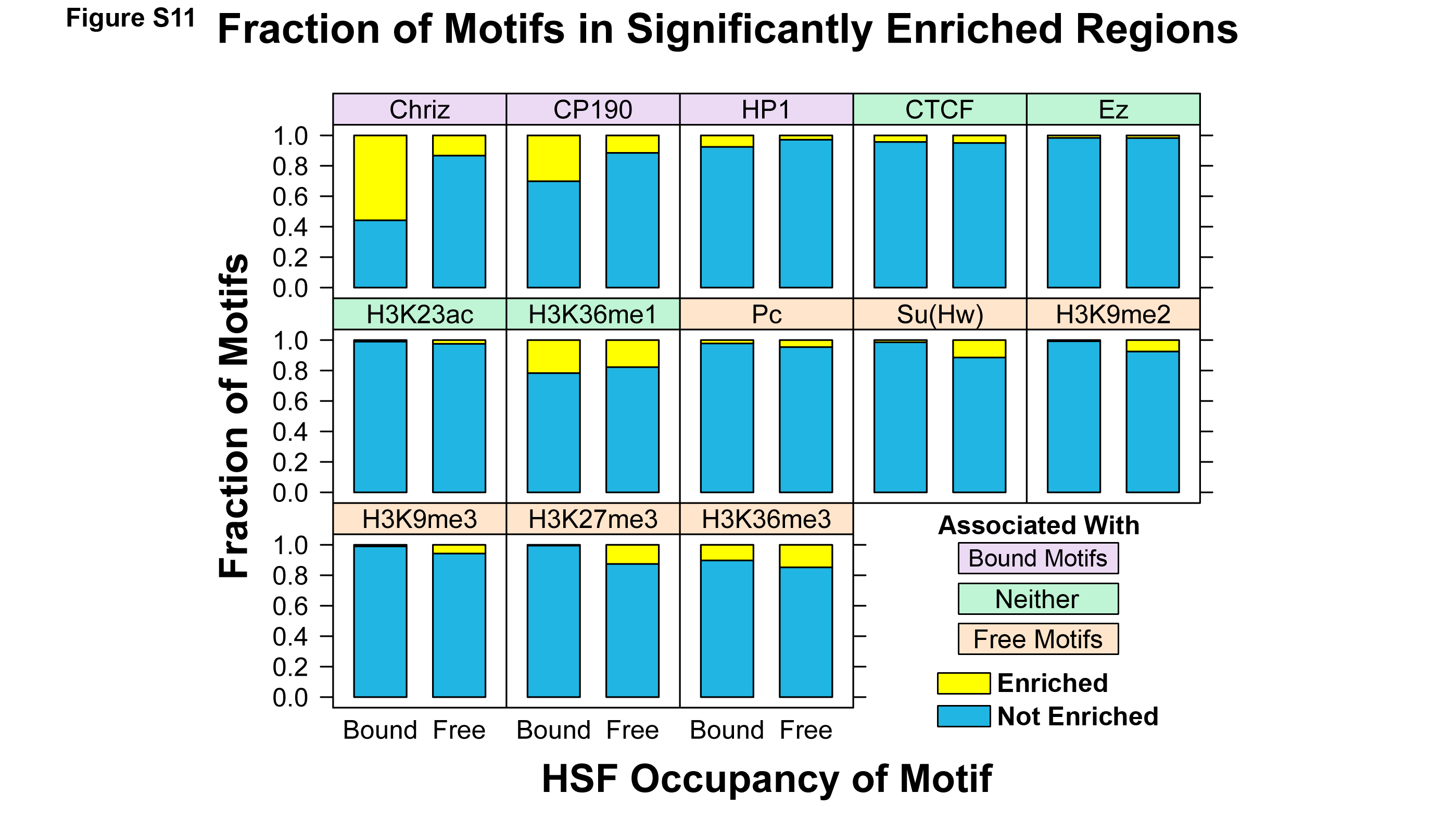

Supplement: Figure S11 — HSF-bound HSE motifs are associated with marks of active chromatin. The fraction of both bound and unbound HSEs in regions of significant enrichment for a given factor or histone modification. Fisher's exact test was used to determine the association between either HSF-bound or HSF-free motifs and each modENCODE factor or histone modification (Table S1). The yellow fraction of the bar chart represents HSF binding sites that are in regions of significant enrichment, while blue depicts all non-enriched sites. A small fraction of HSF-free motifs are statistically associated with marks of repressed chromatin (Polycomb, H3K9me2, H3K9me3, H3K27me3, and H3K36me3). (1.28 MB TIF) [file pgen.1001114.s011.tif]

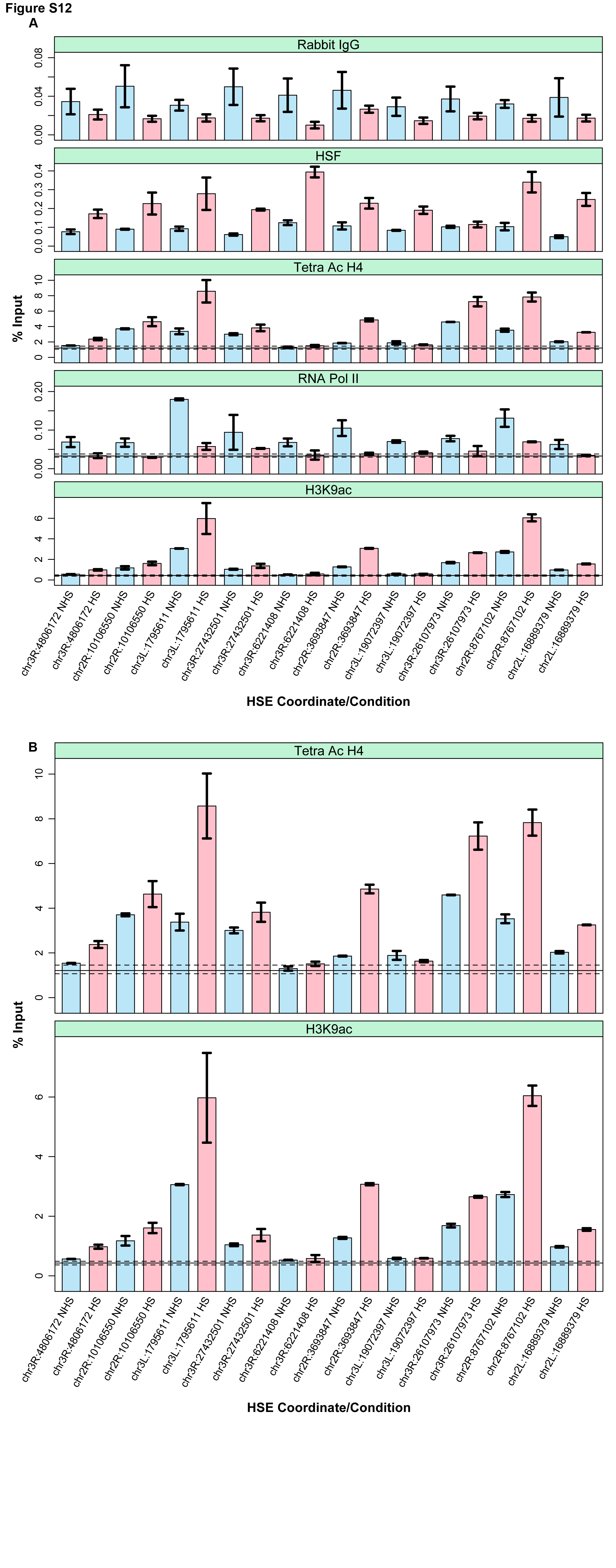

Supplement: Figure S12 — H3K9 acetylation, tetra-acetylated H4 and RNA Pol II are enriched at inducibly bound HSEs in our NHS cell populations. (A) We performed ChIP-qPCR for RNA Pol II, H3K9ac, H4TetraAc and HSF in cells for a subset of HSF-binding sites (Figure S10) that were shown to contain RNA Pol II, tetra-acetylated H4 and H3K9ac in modENCODE experiments. Under NHS conditions, HSF is undetectably bound, but the activation marks are present. The blue and pink bars represent NHS and 20′ HS occupancy, respectively, for each factor. Precipitation with Rabbit IgG controls for non-specific pull-down at this site for each condition (first sub-panel) and dashed and solid lines indicate the range of background intensities for non-specific background pull-down by each antibody (see Materials and Methods) and provides an estimated threshold for assessing enrichment over background. Taken with Figure S10, we conclude that the modENCODE conditions and our conditions are directly comparable. (B) Enlargement of Tetra Ac H4 and H3K9ac plots in panel (A). (2.94 MB TIF) [file pgen.1001114.s012.tif]

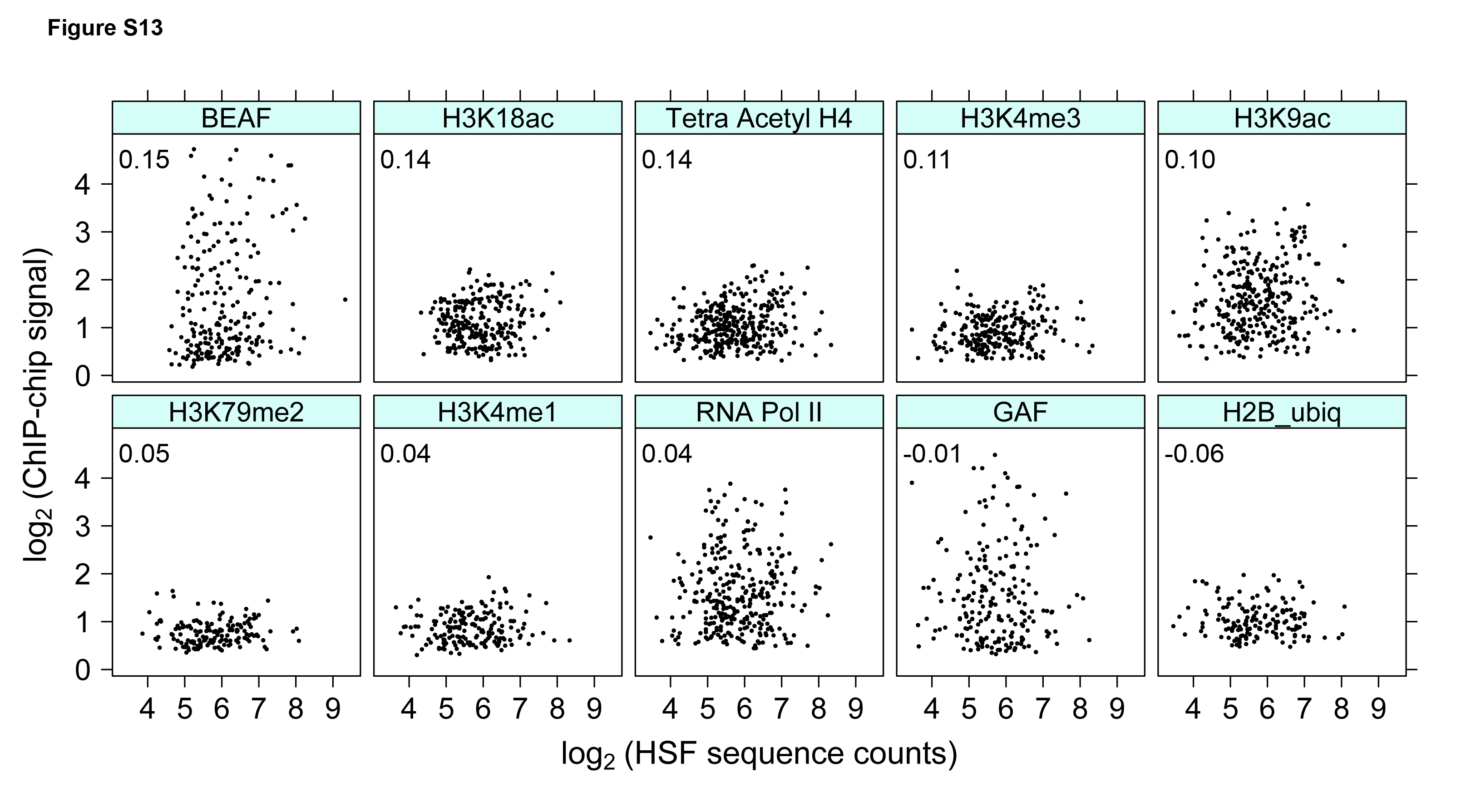

Supplement: Figure S13 — The intensity of activation marks prior to HS modestly correlates with induced HSF binding levels. At those sites that were enriched for a given mark or factor in Figure 4, the modENCODE ChIP-chip intensities for each HSE were correlated to the HSF binding intensity after HS. ChIP-chip intensity is defined as the average microarray intensity of all the probes in a 400 base window centered on the motif. HSF binding intensity is defined as the number of tags whose 5′ ends map in the 240 base window centered on the HSE. The Pearson correlation coefficient is indicated in the top-left of the panel. Only BEAF, tetra-acetylated H4 and H3K18ac had significant correlations with p-values below 0.05. (1.26 MB TIF) [file pgen.1001114.s013.tif]

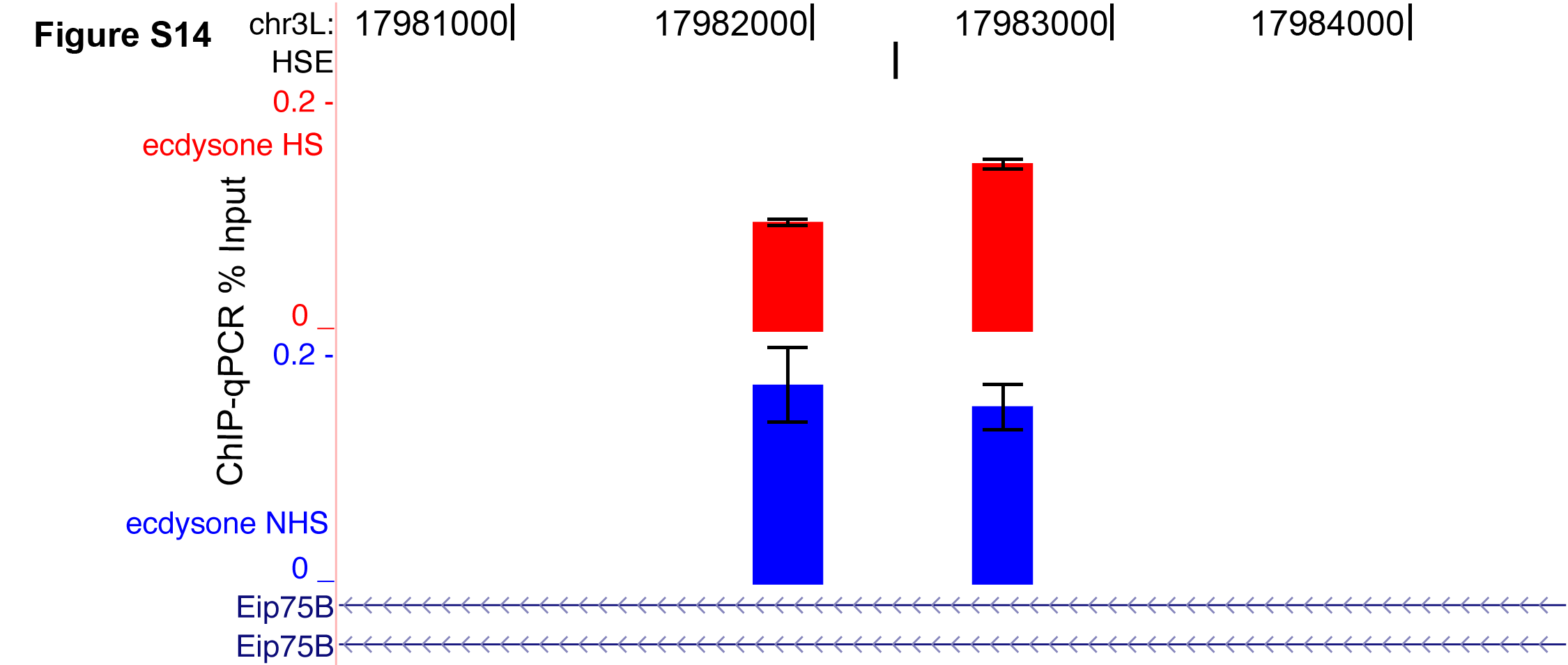

Supplement: Figure S14 — HSF may act as a roadblock to RNA Pol II. We perform RNA Pol II ChIP-qPCR at sites flanking the HSE shown in Figure 6. The widths of the bars span the genomic coordinates of the qPCR amplicons. After ecdysone treatment, there are comparable amounts of RNA Pol II at each site; however, there is a modest depletion of RNA Pol II downstream of the HSE following HS. This finding lends support a model whereby HSF can act as a roadblock to repress transcription. (7.02 MB TIF) [file pgen.1001114.s014.tif]

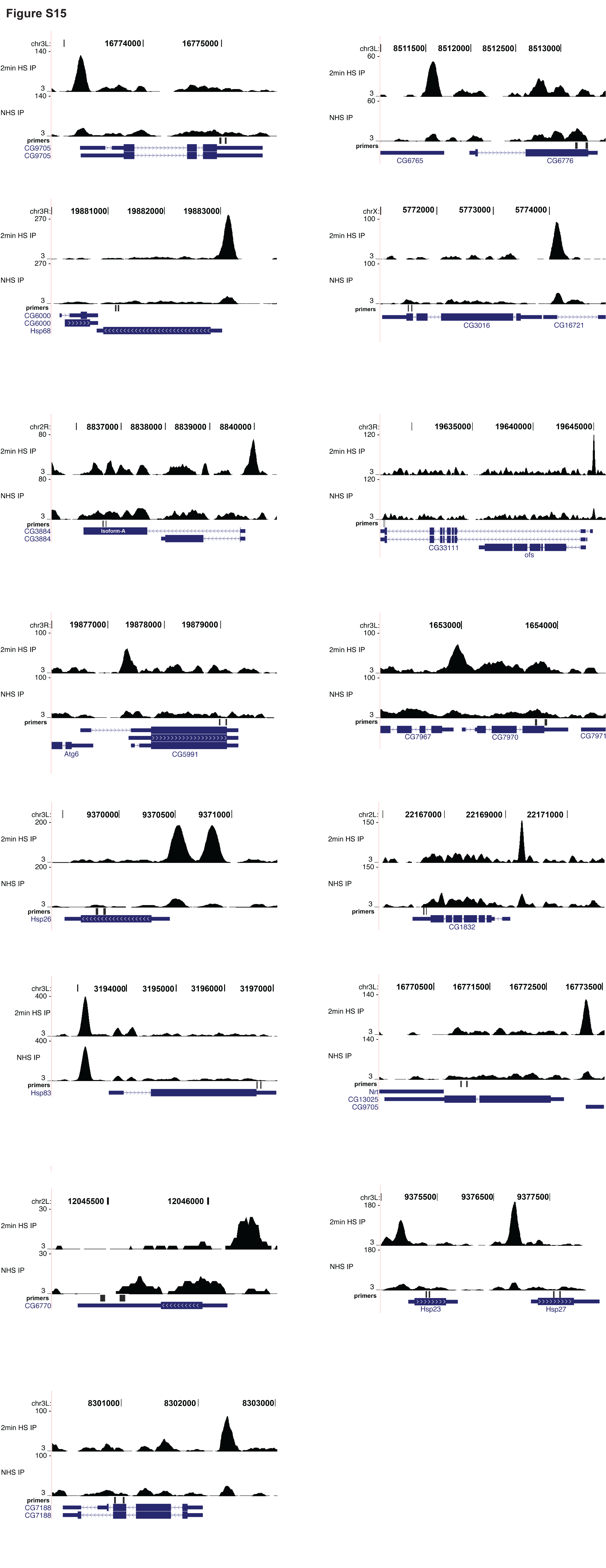

Supplement: Figure S15 — Promoter-bound HSF has varying induction effects. Each gene from Figure 7 is inducibly bound by HSF as early as 2 minutes after heat shock (the top track of each panel). The RT-qPCR assay in Figure 7 was performed after 20 minutes of HS, which allows 18 minutes for mRNA accumulation. The qPCR primers for assaying mRNA levels are illustrated above the gene annotation. (3.89 MB TIF) [file pgen.1001114.s015.tif]

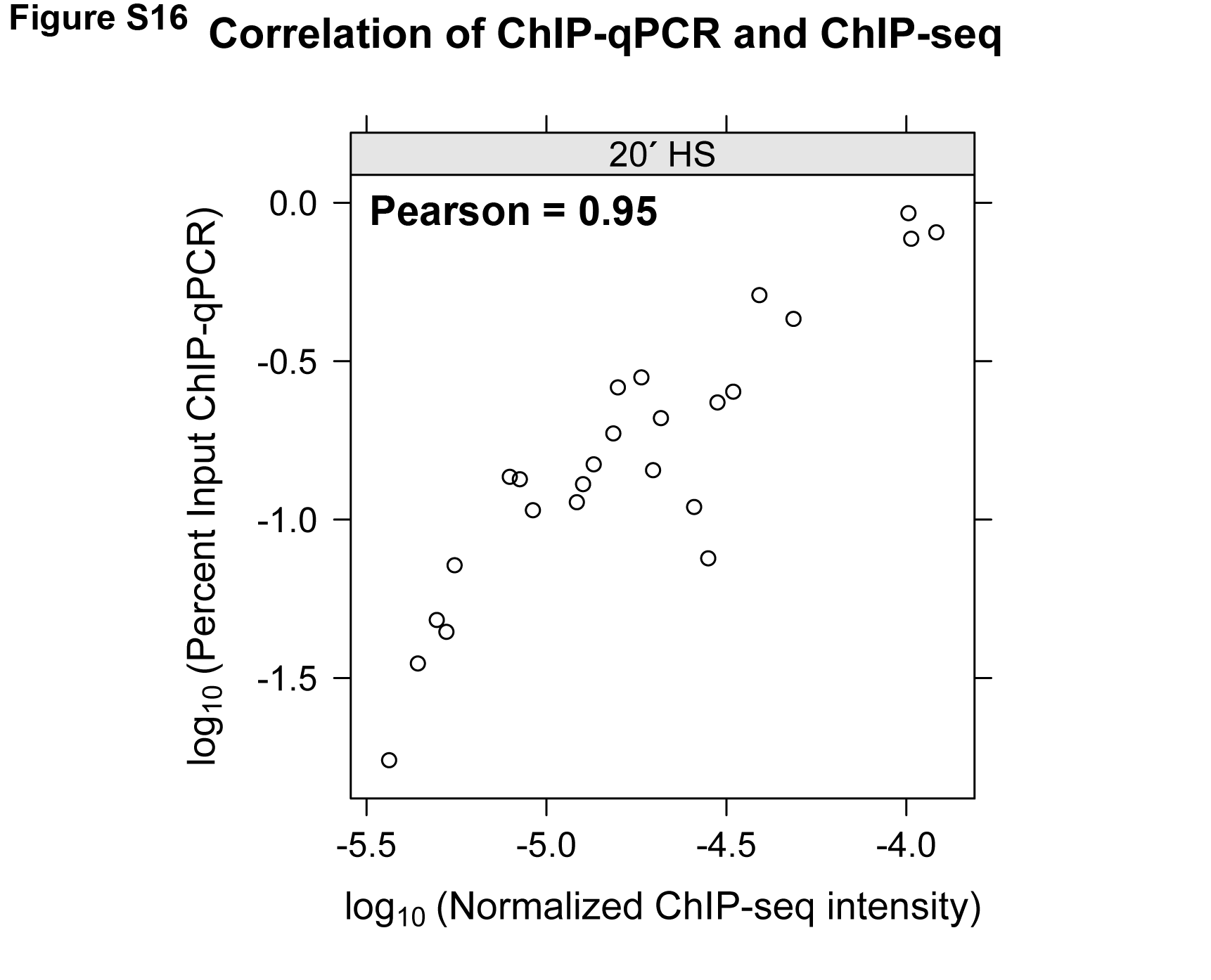

Supplement: Figure S16 — ChIP-seq quantification recapitulates ChIP-qPCR intensities. The ChIP-seq peaks at 25 loci, representing a wide range of intensities, were quantified by normalizing the tag count density in the 320 base window centered on the HSE motif. The corresponding region was quantified by qPCR, using IPed DNA from an independent biological replicate that did not undergo size selection or amplification. The primers for qPCR and the intensities for ChIP-seq and ChIP-qPCR are listed in Table S7. (7.57 MB TIF) [file pgen.1001114.s016.tif]

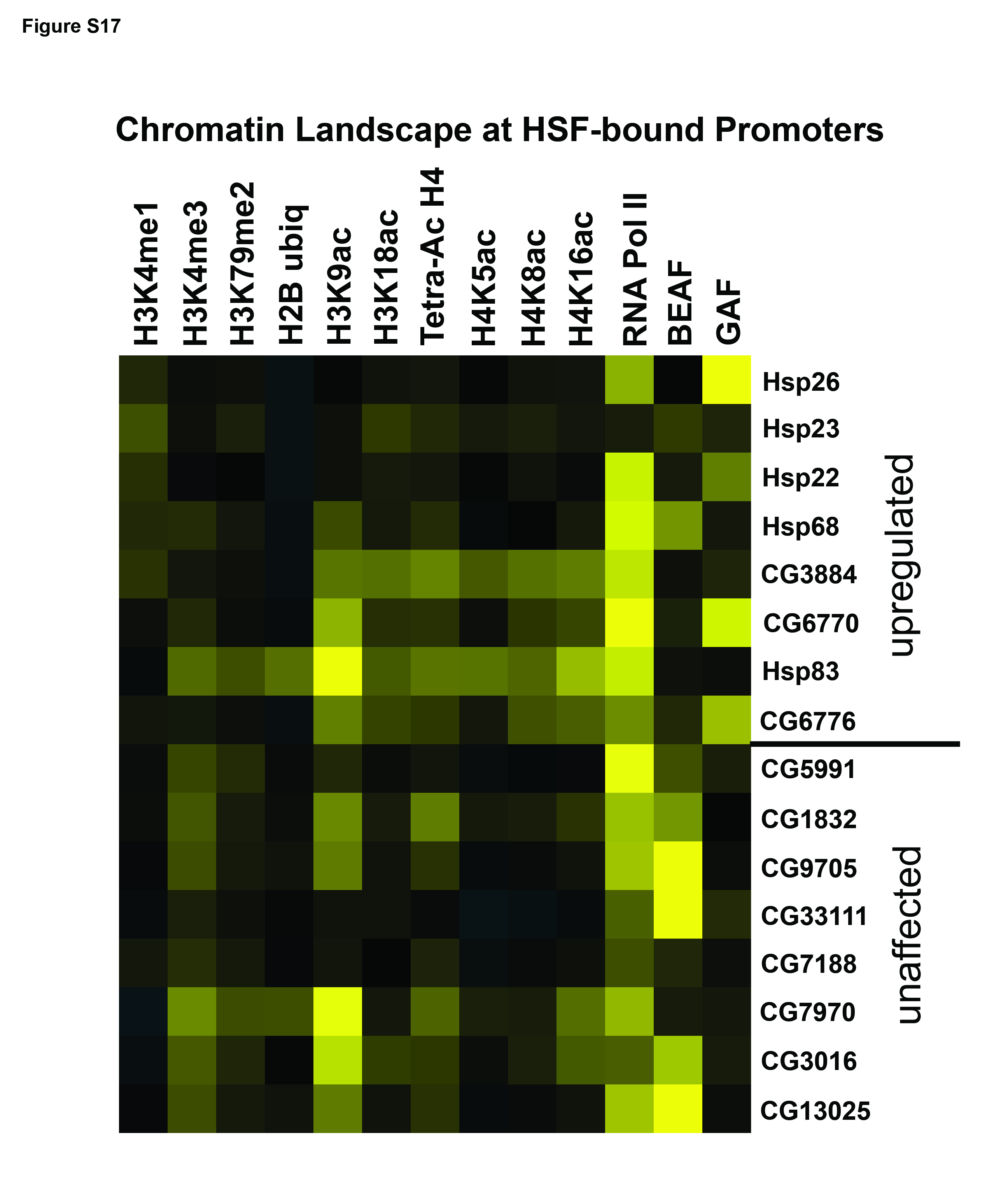

Supplement: Figure S17 — Chromatin landscape at HSF-bound promoters. Each HSF-bound promoter corresponds to an individual row. Rows are arranged from top to bottom by decreasing fold-induction after HS (Figure 7). Columns represent the average microarray intensity of all the probes in a 400 base window centered on the motif for a given factor or histone modification. (2.24 MB TIF) [file pgen.1001114.s017.tif]
